# LTS and FS Inhibitory Interneurons, Short-Term Synaptic Plasticity, and Cortical Circuit Dynamics

**DOI:** 10.1371/journal.pcbi.1002248

**Published:** 2011-10-27

**Authors:** Itai Hayut, Erika E. Fanselow, Barry W. Connors, David Golomb

**Affiliations:** 1 Department of Physiology and Neurobiology and Zlotowski Center for Neuroscience, Faculty of Health Sciences, Ben Gurion University, Be'er-Sheva, Israel; 2 Department of Physics, Faculty of Natural Sciences, Ben Gurion University, Be'er-Sheva, Israel; 3 Department of Neuroscience, Division of Biology and Medicine, Brown University, Providence, Rhode Island, United States of America; 4 Department of Neurobiology, University of Pittsburgh School of Medicine, Pittsburgh, Pennsylvania, United States of America; Université Paris Descartes, Centre National de la Recherche Scientifique, France

## Abstract

Somatostatin-expressing, low threshold-spiking (LTS) cells and fast-spiking (FS) cells are two common subtypes of inhibitory neocortical interneuron. Excitatory synapses from regular-spiking (RS) pyramidal neurons to LTS cells strongly facilitate when activated repetitively, whereas RS-to-FS synapses depress. This suggests that LTS neurons may be especially relevant at high rate regimes and protect cortical circuits against over-excitation and seizures. However, the inhibitory synapses from LTS cells usually depress, which may reduce their effectiveness at high rates. We ask: by which mechanisms and at what firing rates do LTS neurons control the activity of cortical circuits responding to thalamic input, and how is control by LTS neurons different from that of FS neurons? We study rate models of circuits that include RS cells and LTS and FS inhibitory cells with short-term synaptic plasticity. LTS neurons shift the RS firing-rate vs. current curve to the right at high rates and reduce its slope at low rates; the LTS effect is delayed and prolonged. FS neurons always shift the curve to the right and affect RS firing transiently. In an RS-LTS-FS network, FS neurons reach a quiescent state if they receive weak input, LTS neurons are quiescent if RS neurons receive weak input, and both FS and RS populations are active if they both receive large inputs. In general, FS neurons tend to follow the spiking of RS neurons much more closely than LTS neurons. A novel type of facilitation-induced slow oscillations is observed above the LTS firing threshold with a frequency determined by the time scale of recovery from facilitation. To conclude, contrary to earlier proposals, LTS neurons affect the transient and steady state responses of cortical circuits over a range of firing rates, not only during the high rate regime; LTS neurons protect against over-activation about as well as FS neurons.

## Author Summary

The brain consists of circuits of neurons that signal to one another via synapses. There are two classes of neurons: excitatory cells, which cause other neurons to become more active, and inhibitory neurons, which cause other neurons to become less active. It is thought that the activity of excitatory neurons is kept in check largely by inhibitory neurons; when such an inhibitory “brake” fails, a seizure can result. Inhibitory neurons of the low-threshold spiking (LTS) subtype can potentially fulfill this braking, or anticonvulsant, role because the synaptic input to these neurons facilitates, i.e., those neurons are active when excitatory neurons are strongly active. Using a computational model we show that, because the synaptic output of LTS neurons onto excitatory neurons depresses (decreases with activity), the ability of LTS neurons to prevent strong cortical activity and seizures is not qualitatively larger than that of inhibitory neurons of another subtype, the fast-spiking (FS) cells. Furthermore, short-term (∼one second) changes in the strength of synapses to and from LTS interneurons allow them to shape the behavior of cortical circuits even at modest rates of activity, and an RS-LTS-FS circuit is capable of producing slow oscillations, on the time scale of these short-term changes.

## Introduction

Low threshold-spiking (LTS) neurons are a specific subtype of interneuron in the neocortex. Their somata are located in layers 2–6 [1], and they include the Martinotti cells of layer 5 [2], [3], [4], [5] and the green fluorescent protein (GFP)-expressing neurons of the GIN line of transgenic mice [6], [7], [8]. LTS neurons express the neuropeptide, somatostatin, their action potentials have intermediate duration, and they adapt in response to suprathreshold step current injections [9]. The difference between the resting membrane potential and firing threshold of LTS cells is about 12 mV, smaller than observed in excitatory neurons or other types of inhibitory neurons [7]. LTS cells are mutually coupled by electrical synapses [10], but inhibitory chemical synapses between them are only rarely observed [9]. Excitatory synapses from regular-spiking (RS) neurons onto LTS neurons show strong short-term facilitation [7], [9], [11], [12], [13], whereas inhibitory synapses from LTS neurons onto to RS neurons usually depress [7], [9]. LTS neurons are reciprocally coupled by depressing synapses to inhibitory neurons of the parvalbumin-expressing, fast-spiking (FS) type [10], [14]. RS and FS neurons, but not LTS neurons in layer 4, receive thalamic input [10], [15]. There are conflicting data regarding the possibility that LTS neurons in other layers are innervated by thalamocortical axons (see [15], [16]). LTS neurons in layer 3 are excited by sensory inputs during whisking [17]), but these inputs could represent ascending layer 4-to-layer 3 excitation or neuromodulatory pathways.

Because of the strongly facilitating nature of the RS-to-LTS excitatory synapses, rapid stimulation of a few RS neurons or, sometimes, even a single RS neuron can cause LTS neurons to fire spikes [13]. As a result, LTS neurons may mediate disynaptic inhibition between neocortical pyramidal neurons [18], [19], and simultaneous short bursts in four excitatory neurons are sufficient to exert disynaptic inhibition in all neighboring excitatory neurons [20]. When an RS neuron is stimulated and spikes repetitively, this disynaptic inhibition is delayed with respect to the stimulus initiation because RS-to-LTS synapses need time to facilitate before the LTS neuron can fire its own spikes. Based on their experimental results, Beierlein *et al.* [9], Silberberg and Markram [18] and Kapfer *et al.* [19] hypothesized that LTS neurons are important for maintaining the balance between excitation and inhibition in the cortical circuit. Because the amount of excitation varies with the activity of neurons that are presynaptic to cortical neurons (e.g. thalamic relay cells), maintaining this balance is a dynamic process in which LTS neurons may play an important role. For example, when the firing rate of excitatory neurons is high, facilitating excitatory input could generate a supralinear response of LTS neurons and thus prevent overactivation of excitatory neurons (i.e., activation beyond what is normal, leading to pathological behavior). This could protect the cortical network against seizures. Consistent with the idea that LTS cells serve a protective function is the observation that selective loss of somatostatin-positive dendritic-targeting interneurons (cells similar to neocortical LTS neurons) in hippocampus correlates with epileptic states [21], [22]. More recently, it was suggested that LTS neurons balance excitation and prevent runaway cortical activity by decreasing the gain of pyramidal cell output [23].

The ability of LTS neurons to protect against network over-activation may be limited, however, by the depressive nature of LTS-to-RS inhibitory synapses. Furthermore, short-term synaptic plasticity can lead to firing patterns more complex than stable firing rates. The existence of two time-scales in the system dynamics — the fast time-scale of the AMPA receptor- and GABA_A_ receptor-mediated postsynaptic potentials (PSPs), and the slow time-scale of synaptic depression and facilitation processes — may, in principle, lead to various types of network oscillations or more complicated patterns. Such network oscillations were observed in previous models of excitatory and inhibitory neurons [24], [25], [26], [27], but those models did not take into account the specific physiological characteristics of LTS neurons.

In this study we ask: by which mechanisms and at what firing rates do LTS neurons control the activity of cortical circuits responding to thalamic input, and how is control by LTS neurons different from that of FS neurons? To be more specific, we compare the dynamical behavior of LTS neurons with those of FS neurons in networks with only one type of inhibitory interneuron and in networks with both inhibitory populations, to address the hypothesis of Beierlein *et al.* [9], Silberberg and Markram [18] and Kapfer *et al.* [19]. We consider a rate model of cortical networks [28], [29], [30] that includes RS, LTS and FS neurons with short-term synaptic plasticity [31], [32], and study its responses to external inputs.

## Results

### Model Description

#### Circuit architecture

**Figure 1:**
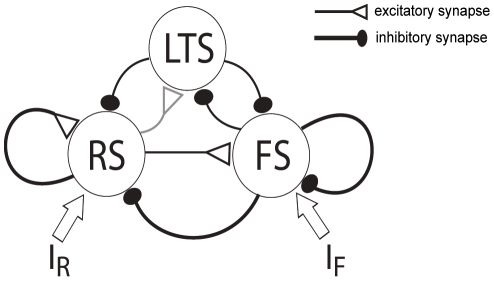
Schematic architecture of the RS-LTS-FS cortical circuit. Open triangles denote excitatory synapses, and solid ellipses denote inhibitory synapses. Black lines denote depressing synapses, and grey lines denote facilitating synapses.

The architecture of the full RS-LTS-FS cortical network, based on [9], [10], is shown in Figure 1. RS neurons excite RS, LTS and FS neurons. FS neurons inhibit RS, LTS and FS neurons. LTS neurons inhibit RS and FS neurons, but not LTS neurons. In this article, we focus on the short-term plasticity of chemical synapses between cortical neurons, and therefore assume three simplifications. First, we use firing rate models and effectively average over the spiking dynamics of neurons [29], [31], [33], [34]. Second, we do not consider electrical synapses between cortical interneurons [1], [10]. Third, we assume constant or step external input, and do not take into account depression or facilitation of thalamocortical synapses [35].

RS and FS neurons [15], [36], but not LTS neurons in layer 4 [9] receive external thalamic input. Whether LTS neurons in other layers are innervated by the thalamus still remains unresolved (see [15] vs. [16], [17]). Therefore, we initially study a model in which LTS neurons do not receive thalamic input, and analyze the effects of thalamic input onto LTS neurons separately. In addition, LTS neurons are activated by various neuromodulators [7]. This effect is modeled as a reduction of the LTS threshold.

We examine the model in four stages. First, we consider a network of RS and LTS neurons, where RS neurons receive external inputs (either step or absence-seizure-like). Second, we study an RS-FS network to demonstrate the differences between the effects of the FS and LTS populations on the circuit. Third, we consider a full network composed of RS, LTS and FS neuronal populations. Finally, we analyze a slow oscillation state emerging from this network.

#### Synaptic dynamics and neuronal firing rates

Our technical approach makes use of the formulation of Shriki *et al.* for rate equations [28], [29], [30]. Each neuronal population is described by its firing rate *M* with a subscript *i* denoting the population: *R* for RS, *F* for FS and *L* for LTS. A synaptic connection from a neuron from population *j* to a neuron from population 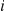 is characterized by three dynamic variables with the subscripts *ij*: the fraction of open synaptic channels *s*, the running fraction of vesicles available for release *x*, and the running value of the “utilization” parameter *u* [31], [32]. The variable *u* quantifies the conditional probability of release of a vesicle in response to an action potential arriving to the presynaptic terminal, assuming that vesicle is ready for release before the spike arrives. Each synaptic connection is characterized by a set of five parameters: the efficacy *g*, the initial conditional probability of release *U*, assuming that a previous presynaptic spike has not occurred for a long time, the decay time of the post-synaptic current *τ_s_*, and the recovery time constants from facilitation and depression, *τ_f_* and *τ_r_* respectively. The dynamics for each synaptic connection are therefore described by the following equations:
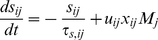
(1)

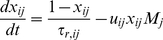
(2)

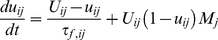
(3)The firing rates *M_i_* for the three neuronal populations are determined according to the circuit diagram (Figure 1):
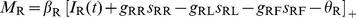
(4)

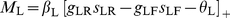
(5)

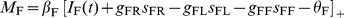
(6)where, for each population, *I_i_*(*t*) is the external input from sources outside of the local cortical network, *θ_i_* is the neuronal threshold, and *β_i_* is the neuronal gain calculated according to the *f*-*I* curve at steady state [8], [9]. The coefficients of synaptic conductances are denoted by *g_ij_*, and the total synaptic input from neuronal population *j* to a neuron from population *i* is *g_ij_ s_ij_*. The function []_+_ is the rectification (linear-threshold) function: [*x*]_+_ = *x* for *x*≥0 and [*x*]_+_ = 0 otherwise. Note that the currents *I*_i_ and the conductances *g_ij_* are measured in arbitrary units [30].

#### Model parameters

Despite the fact that our model is relatively simple, it includes many parameters. Therefore, it is important to consider ranges of biophysical parameters. It is, of course, impossible to study the entire multidimensional space of parameters. We limit the range of parameters by taking most of their values from the literature, but some of them remain unknown. In particular, the maximal synaptic conductances a neuron receives from its presynaptic neurons are often hard to determine. Knowing these difficulties, we use the following strategy that we have often used in the past (e.g., [37]). We choose a biophysically plausible parameter set as a reference point in the parameter space. The reference parameter values for the model are written in Tables 1 and 2 (see Methods). Starting from this point, we vary one or two parameters to study their effect. Specifically, we study sub-networks of RS-LTS and RS-FS populations to investigate the respective role of the two types of interneurons before studying the full RS-LTS-FS network. Exploring the dependence on parameters provides us with an understanding of the different dynamical patterns the network can exhibit.

**Table 1:** Reference parameters for the neuronal populations, based on [7].

Neuronal population	*θ* (nA)	*β* (ms^−1^ nA^−1^)
RS	0.1	0.11
LTS	0.05	0.32
FS	0.28	0.35

**Table 2:** Reference parameters for the synapses between the various types of neurons.

Synaptic connection	*τ_s_* (ms)	*τ_f_* (ms)	*τ_r_* (ms)	*U*	*g*	Reference
RS←RS	2	0	463	0.21	5	[83]
RS←LTS	6.3	0	1250	0.3	35	[18]
LTS←RS	2	670	0	0.09	7	[18]
RS←FS	2	0	875	0.14	38	[47]
FS←RS	2	0	227	0.3	18	[47], [84]
FS←LTS	2	0	400	0.3	5	
LTS←FS	2	0	400	0.3	10	
FS←FS	2	0	400	0.3	20	

### RS-LTS Networks without RS-to-RS Recurrent Connections

We consider a network of two populations, composed of RS and LTS neurons. To explore the role of RS-to-LTS and LTS-to-RS synapses, our first step is to study a model with these synaptic connections only, and the effect of the RS-to-RS synapses will be studied later. RS-to-LTS synapses facilitate (*τ_f_*_,LR_ = 670 ms) and LTS-to-RS synapses depress (*τ_r_*_,RL_ = 1250 ms) [18] (see Methods and Table 2). Therefore, *x*_LR_ = 1, *u*_RL_ = *U*_RL_, and equations 1–3 for the RS-LTS system become
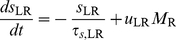
(7)

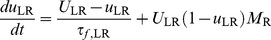
(8)

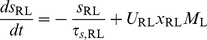
(9)

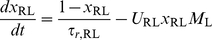
(10)


#### Steady-state firing

**Figure 2:**
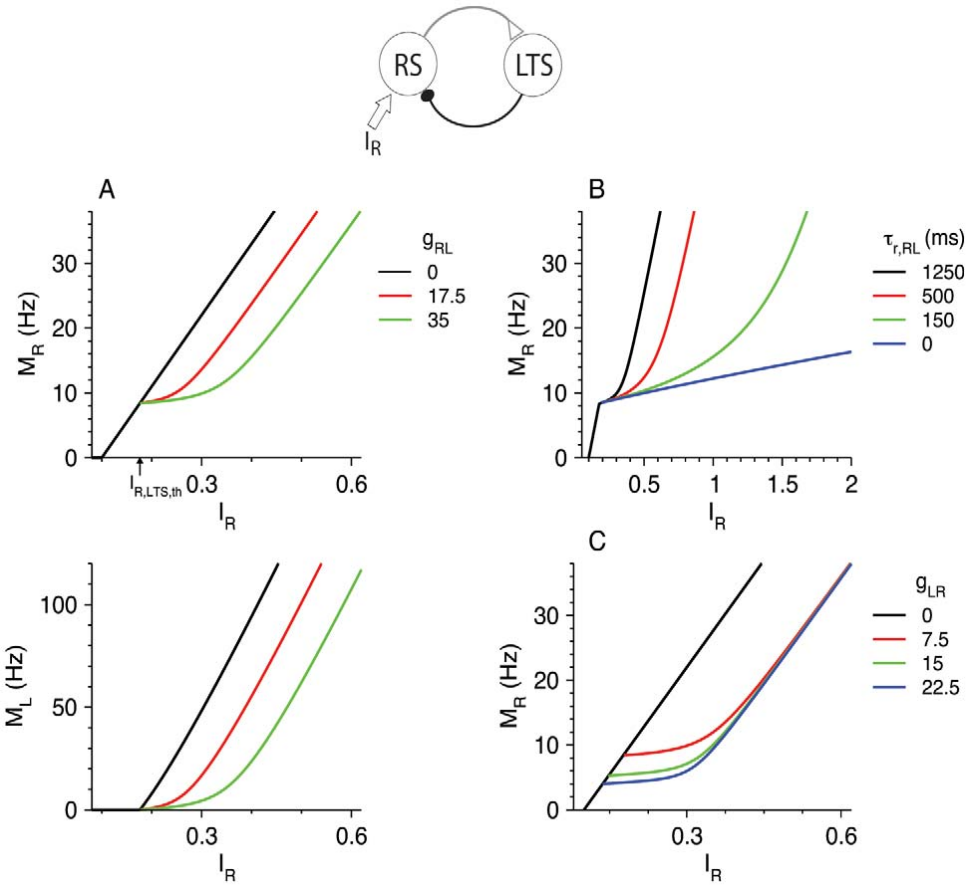
Steady-state response of the RS-LTS network with *g*_RR_ = 0 to constant inputs to the RS neurons. (A) *M*_R_-*I*_R_ curves (top panel) and *M*_L_-*I*_R_ curves (bottom panel) are plotted for *g*_RL_ = 0 (black), 17.5 (red) and 35 (green). Additional parameters are *τ_r_*_,RL_ = 1250 ms, g_LR_ = 7.5. The arrow below the abscissa in the top panel points to the value of *I*_R,LTS,th_. (B) *M*_R_-*I*_R_ curves are plotted for *τ_r_*_,RL_ = 1250 ms (black), 500 ms (red) and 150 ms (green) and 0 (blue). Additional parameters are *g*_RL_ = 35, g_LR_ = 7.5. (C) *M*_R_-*I*_R_ curves are plotted for g_LR_ = 0 (black), 7.5 (red), 15 (green) and 22.5 (blue). Additional parameters are *g*_RL_ = 35, *τ_r_*_,RL_ = 1250 ms.

When the input to the RS population, *I*_R_, is constant in time, the steady-state values of the system are
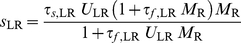
(11)

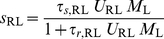
(12)where
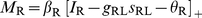
(13)and
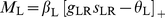
(14)The firing rates of the two populations, *M*_R_ and *M*_L_, as functions of *I*_R_ for several values of the LTS-to-RS synaptic conductance coefficient *g*_RL_ are shown in Figure 2A. When *g*_RL_ = 0, the RS population is silent for *I*_R_≤*θ*_R_, and *M*_R_ increases linearly with *I*_R_−*θ*_R_ for *I*_R_>*θ*_R_. LTS neurons fire for *I*_R_>*I*_R,LTS,th_ (*I*_R,LTS,th_>*θ*_R_), (“Threshold for LTS firing for *g*_RR_ = 0” in Methods, Equations 22,23), and inhibit RS neurons for *g*_RL_>0. For *I*_R_ just above *I*_R,LTS,th_, *M*_L_ is small and *M*_R_ increases only weakly with *I*_R_. Since *τ_r_*_,RL_ *U*_RL_ *M*_L_<<1, equation 12 becomes 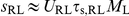, and *dM*_R_/*dI*_R_ just above *I*_R,LTS,th_ is (Equations 24,25)
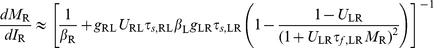
(15)
*i.e.*, the slope *dM*_R_/*dI*_R_ at threshold scales like 1/*g*_RL_ for large *g*_RL_.

For large input *I*_R_, the firing rates *M*_R_ and *M*_L_ are large as well, and *s*_RL_≈*τ_s_*_,RL_/*τ_r_*_,RL_ (Equation 12). Using equation 13, we obtain
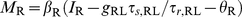
(16)Therefore, *M*_R_ increases linearly with *I*_R_ with a slope (gain) *β*_R_, and is reduced by inhibition by a constant value *β*_R_ *g*_RL_ *τ_s_*_,RL_/*τ_r_*_,RL_. Like *M*_R_, *M*_L_ increases linearly with *I*_R_ for large *I*_R_: 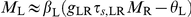 (Figure 2A). The gains of *M*_R_ and *M*_L_ with *I*_R_ remain relatively small in an *I*_R_ range of about *g*_RL_ *τ_s_*_,RL_/*τ_r_*_,RL_, before they reach approximately their maximal values.

The reduction of activity by a constant value at large *I*_R_ (and large firing rates) is a result of the properties of the depressing LTS-to-RS synapses at high firing rates *M*_L_. The postsynaptic current (PSC) amplitude for such a synapse is inversely proportional to *M*_L_, the firing rate of the presynaptic neuron [38], and therefore the total LTS-to-RS inhibition is independent of *M*_L_. This constant inhibition shifts the *M*_R_-*I*_R_ curve to the right by a fixed value, and this shift is translated to a constant reduction of *M*_R_ because of the linear dependency of *M*_R_ on the total input to the neuronal population. Indeed, the inhibitory effect on the *M*_R_-*I*_R_ curve is enhanced when *τ_r_*_,RL_ is small and LTS-to-RS neurons recover faster from depression (Figure 2B). Just above *I*_R,LTS,th_, the slope of the *M*_R_-*I*_R_ curve does not depend on *τ_r_*_,RL_ because the neurons hardly depress for small *M*_L_. When *τ_r_*_,RL_ = 0 (no depression), the slope of *M*_R_-*I*_R_ curve is always smaller than *β*_R_ when the LTS neurons fire. Increasing the RS-to-LTS excitatory conductance *g*_LR_ reduces *I*_R,LTS,th_ but does not affect the value of *M*_R_ at large *M*_R_ (and therefore *I*_R_) values (Figure 2C).

#### Dynamics of firing response to step inputs

**Figure 3:**
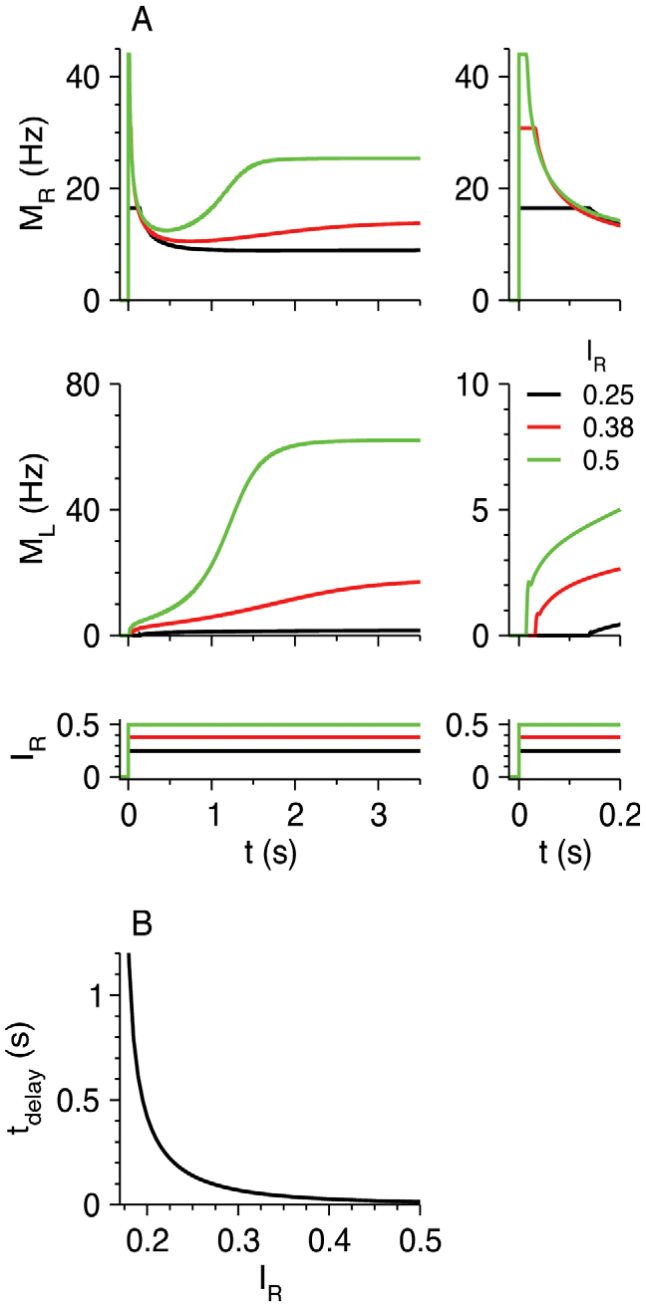
Response of the RS-LTS network with *g*_RR_ = 0 to step inputs *I*_R_Θ(*t*) to the RS neurons. Additional parameters are *g*_RL_ = 35, g_LR_ = 7.5. (A) Time courses of *M*_R_ (top panels) and *M*_L_ (middle panels) for *I*_R_ = 0.25 (black), 0.38 (red) and 0.5 (green)(bottom panels). The right top and middle panels depict the time course of *M*_R_ and *M*_L_ in a shorter time scale to emphasize the delay to the onset of LTS activity. (B) The delay time *t*_delay_ to the onset of firing of LTS neurons as a function of *I*_R_. The *t*_delay_ values computed from simulations are almost indistinguishable from those computed from Equation 28.

We consider step inputs to the RS population starting at time *t* = 0 with amplitudes *I*_R_, *I*_R_Θ(*t*) (Θ being the Heaviside function). Temporal profiles of the firing response of the RS and LTS population to those inputs are shown in Figure 3A. For a just-suprathreshold input *I*_R_, LTS neurons start to fire after a delay *t*_delay_ (“Delay of LTS firing in response to step input” in Methods), because RS-to-LTS synapses need time to facilitate and excite LTS cells. The dependence of *t*_delay_ on *I*_R_, computed both from simulations and from Equation 28 (Methods), is shown in Figure 3B. The time *t*_delay_ diverges logarithmically as *I*_R_ approaches *I*_R,LTS,th_ from above, and is small, on order *τ_s_*_,LR_, when *I*_R_ is much larger than *I*_R,LTS,th_. After LTS neurons are recruited, they inhibit RS neurons, and this inhibition is stronger for larger *I*_R_ (Figure 3A). For even longer times (and levels of inhibition that are not weak), LTS-to-RS inhibition depresses, and *M*_R_ rebounds, whereas *M*_L_ continues to grow toward its steady-state value.

### Effects of the Extensions to the Model

#### Tonic thalamic or neuromodulatory input to LTS neurons

**Figure 4:**
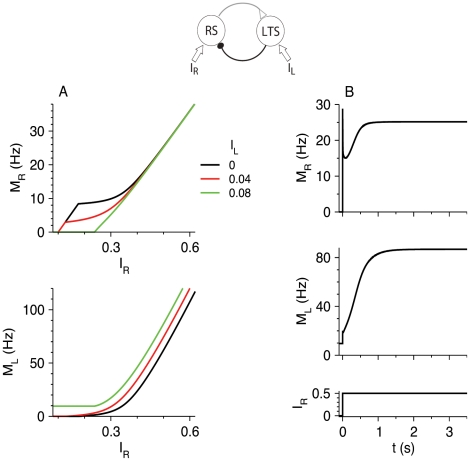
Response of the RS-LTS network with input *I*_L_>0 to LTS neurons. Additional parameters are *g*_RL_ = 35, g_LR_ = 7.5, *g*_RR_ = 0. (A) *M*_R_-*I*_R_ curves (top panel) and *M*_L_-*I*_R_ curves (bottom panel), representing the steady-state response of the circuit to the inputs *I*_R_ and *I*_L_ to the RS and LTS neurons respectively, are plotted for *I*_L_ = 0 (black), 0.04 (red) and 0.08 (green). (B) Response of the circuit to a step input *I*_R_Θ(*t*) to the RS neurons. Time courses of *M*_R_ (top panel) and *M*_L_ (middle panel) are plotted for *I*_R_ = 0.5 (bottom panel). The current *I*_L_ = 0.08 remains constant.

According to some experimental studies, LTS neurons in layers 2–3 or 5 receive thalamic input [16] [17] (see also [15]). Furthermore, LTS neurons may be tonically active in response to the application of various neuromodulators [7], or perhaps from inputs originating in distant cortical areas. Therefore, we study the response of the RS-LTS network when LTS neurons are active in response to tonic thalamic input or neuromodulators, mimicked in the model by introducing external input *I*_L_ to the LTS neurons. This is equivalent to reducing the threshold *θ*_L_. At steady state, the current *I*_L_ reduces *I*_R,LTS,th_ (Figure 4A). At high rates, the activity of RS neurons is not affected by *I*_L_, and the activity of LTS neurons is increased by *β*_L_ *I*_L_. The temporal response of the RS and LTS neurons in the circuit to step current for *I*_L_<*θ*_L_ is similar to the response for positive *I*_L_>*θ*_L_ (*cf.* Figures 3A and 4B), except that *M*_L_ starts from a positive value in the second case.

#### Spike-frequency adaptation

The parameters *θ_i_* and *β_i_* (Equations 4–6) are calculated according to the *f*-*I* curve of the neurons at steady state, but spike frequency adaptation is not considered explicitly in our model. To assess the adaptation effects on the cortical circuit responses, we model adaptation in each neuronal population by introducing an adaptation current variable *a_i_* for each neuronal population *i* [39], [40], which evolves according to the differential equation
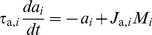
(17)where *τ*_a,*i*_ and 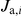 are the adaptation time constant and the adaptation strength constant of the *i*th neuronal population respectively. The firing rates *M_i_* (Equations 4–6) are
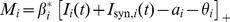
(18)where *I*_syn,*i*_(*t*) is the total synaptic current the neuron receives from the other neurons within the circuit and 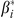 is the neuronal gain of the model with adaptation for *a_i_* = 0. At steady state, 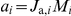, and, with *I*_syn_ = 0, the slope of the *f*-*I* curve is (Equation 18) [40]

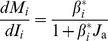
(19)Therefore, to keep the slope of the *f*-*I* curve equal in the models without and with adaptation, we set 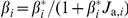. In response to a step function, the initial slope of the *f*-*I* curve (*a_i_* = 0) is *β_i_*, and it decreases to 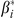 at large times after the stimulus onset (i.e., it is reduced by a factor 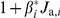). Therefore, based on the values of *β_i_* from Table 1 and Figure 1C in [9], we find: 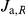 = 2, 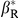 = 0.33, 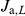 = 1, 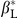 = 0.64.

**Figure 5:**
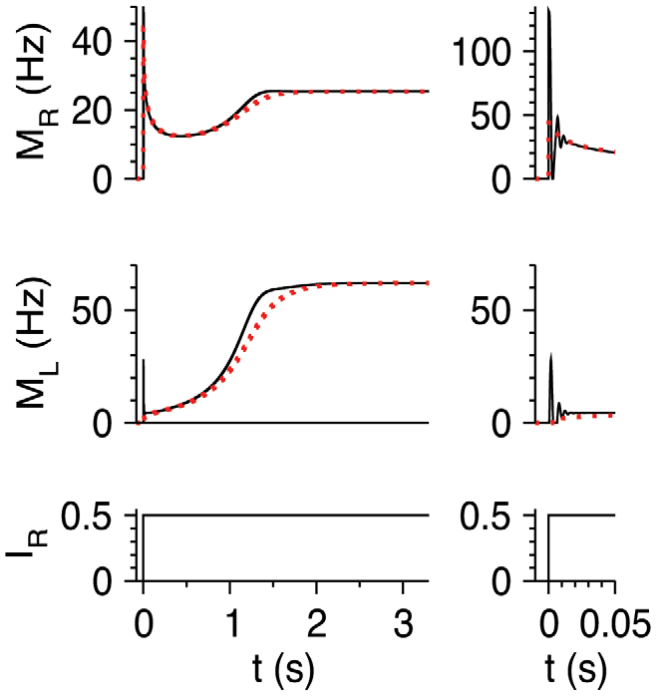
Effects of spike-frequency adaptation. The response of the RS-LTS network to step inputs *I*_R_Θ(*t*) to the RS neurons is shown by plotting the time courses of *M*_R_ (top panels) and *M*_L_ (middle panels) for *I*_R_ = 0.5 (bottom panels). Solid black lines represent the model with adaptation and dotted red lines represent the model without adaptation. The right top and middle panels depict the time course of *M*_R_ and *M*_L_ in a shorter time scale to emphasize the initial response to stimulus onset. Additional parameters are *g*_RL_ = 35, g_LR_ = 7.5, *g*_RR_ = 0.

The relation between *β_i_*, 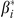 and 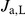 holds as long as the total current *I_i_+I*_syn,*i*_ is constant in time, namely at steady state. This means that the *M_i_*-*I_i_* curve obtained in the model without adaptation (e.g., Figure 2) remains exactly the same when adaptation is introduced, as along as the isolated single cells in the two models have the same *f*-*I* curves. The dynamical response to time-varying stimuli, however, may be modified because the initial response to input is stronger. Indeed, Figure 5 shows that the initial response to a step stimulus of the RS-LTS model with adaptation is stronger, and the model reaches steady state a little bit faster. Except for these differences, the dynamical responses of the model with and without spike-frequency adaptation are very similar.

#### Firing-rate saturation

Neurons exhibit refractoriness and their firing rates saturate and do not diverge in response to strong depolarizing inputs. We explore saturation effects in Supplementary Information Text S1 and Figure S1. Whereas saturation affects the activity at high rates, we find that the contribution of LTS neurons in preventing the circuit from reaching the over-activated regime is qualitatively similar without and with saturation.

#### RS-LTS networks with RS-to-RS recurrent connections

We analyze the effects of RS-to-RS depressing excitatory synapses with a strength *g*_RR_ on the response of RS-LTS circuits to thalamic inputs effects in Supplementary Information Text S1 and Figures S2, S3, S4. If *g*_RR_ is large enough, the system exhibits a stable rest state only if the firing rate *M*_R_ is larger than a critical firing rate *M*_R,c_. Therefore, as happens without depression [41], RS neurons cannot fire at very low rates. At high rates, RS-to-RS connections increase the firing rate by the term 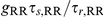. If *g*_RR_ is strong enough, it may induce fast network oscillations with frequencies about 20–60 Hz, which may either be stopped by synaptic depression or be an attractor. During these fast oscillations, LTS neurons are active only when RS neurons are active, i.e. the two populations fire nearly in phase.

### Cortical Response in an Absence-Seizure State

**Figure 6:**
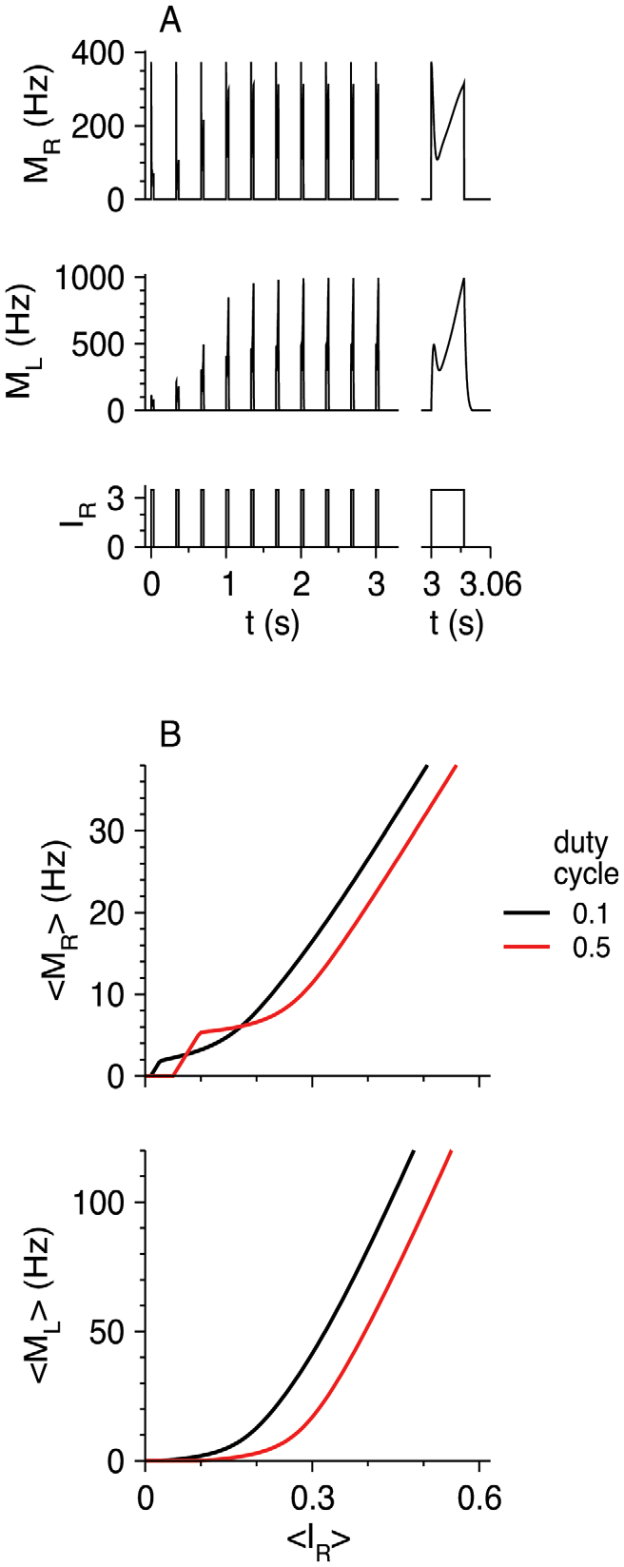
Response of the RS-LTS circuits to absence-seizures input from the thalamus. Additional parameters are *g*_RL_ = 35, g_LR_ = 7.5, *g*_RR_ = 0. (A) Response of the circuit to a periodic square input to the RS neurons, mimicking thalamic input during an absence-seizure state (bottom panel). Time courses of *M*_R_ (top panel) and *M*_L_ (middle panel) are plotted for *I*_R_ with amplitude 3.5 and duty cycle of 0.1 (i.e., <*I*_R_> = 0.35). The right top and middle panels depict the time course of *M*_R_ and *M*_L_ in a shorter time scale to emphasize the temporal form of steady-state activity. (B) <*M*_R_>-<*I*_R_> curves (top panel) and <*M*_L_>-<*I*_R_> (bottom panel), representing the steady-state response of the circuit to absence-seizure-like thalamic input to the RS neurons, are plotted for duty cycles of the active phase of 0.1 (black) and 0.5 (red).

Absence seizures are a type of epilepsy that is considered to originate from the thalamus or at least to be driven by thalamic input [42], [43]. Such seizures are characterized by periodic thalamic input to cortex with a frequency of about 3 Hz or somewhat higher [43], [44], [45], and a duty cycle of the active phase of each thalamic cycle that is larger than 0.1 [46]. To investigate the response of the RS-LTS circuits to such thalamic inputs, we stimulate RS neurons by square-wave periodic input (Figure 6A). Both RS and LTS neurons respond to the onset of each cycle by a brief elevation of their *M* followed by a deep decrease in activity and then more prolonged rebound. The integrated responses of *M*_R_ and *M*_L_ over a cycle (Figure 6A) increase with time towards their steady-state values, which are reached within about 1 sec. This behavior is similar to the evolution of *M*_R_ and *M*_L_ to step inputs (Figure 3). To characterize the properties of the steady-state response to the periodic, absence-seizure-like input, we define the time-averaged value 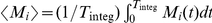, calculated for a large integration time *T*_integ_ after the system has converged to an attractor. Similarly, we define 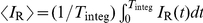. The values of <*M*_R_> and <*M*_L_> as functions of <*I*_R_> are shown in Figure 6B for two values of the duty cycle of the active phase, 0.1 and 0.5 (note that the amplitude of *I*_R_ during the active phase decreases with the duty cycle, to keep <*I*_R_> fixed). In both cases, the steady-state dependencies of <*M*_R_> and <*M*_L_> on <*I*_R_> resemble those of *M*_R_ and *M*_L_ on *I*_R_ for constant stimuli (Figure 2). In particular, these curves become straight lines at high rates with slopes *β*_R_ and *β*_L_ respectively, and are shifted to the left by LTS-to-RS inhibition. As the duty cycle of the active phase of the input is reduced, the value *I*_R,LTS,th_ in which LTS neurons start to fire decreases because the amplitude of the input during that active phase increases. We conclude that the RS-LTS circuit responds to absence-seizure inputs and constant thalamic inputs in qualitatively similar ways.

### RS-FS Networks

To characterize the difference between the roles of FS and LTS neurons in the cortical circuit, we examine a network composed of RS and FS neurons. The RS-FS network is qualitatively different from the RS-LTS network in three respects [5], [9], [47]. First, RS-to-FS excitatory synaptic connections depress whereas RS-to-LTS connections facilitate. Second, FS neurons, but not LTS neurons, receive thalamic input. Third, FS neurons are mutually coupled by chemical synapses. We analyze the response of RS-FS circuits to constant and step inputs.

#### Steady-state firing

**Figure 7:**
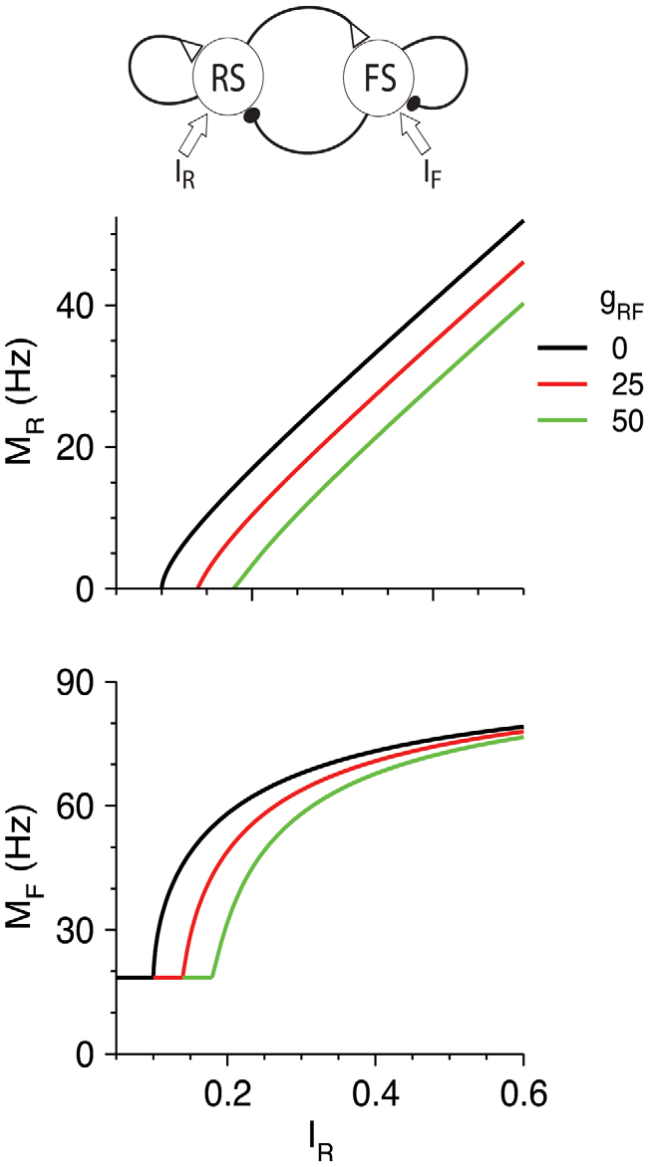
Steady-state response of the RS-FS network to constant inputs to the RS and FS neurons. *M*_R_-*I*_R_ curves (top panel) and *M*_F_-*I*_R_ curves (bottom panel) are plotted for *g*_RF_ = 0 (black), 25 (red) and 50 (green). Additional parameters are: *g*_RR_ = 20, *g*_FR_ = 25, *g*_FF_ = 5, *I*_F_ = 0.35 (independent of *I*_R_).

The steady-state *M*-*I*_R_ curves of the RS and FS populations are shown in Figure 7. FS neurons fire even for *I*_R_<*θ*_R_, and their firing rate increases as RS neurons fire for *I*_R_>*θ*_R_. For large *I*_R_, and therefore large *M*_R_, 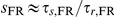, the firing rate *M*_F_ approaches a constant limiting value, *M*_F,max_, that is a solution of the implicit equation (Equations 6,1,2):
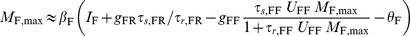
(20)The variable *s*_RF,max_ for large *I*_R_ (Equation 1,2) is 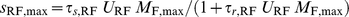, and
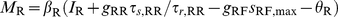
(21)Inhibition from FS neurons, like that from LTS neurons, reduces the steady-state firing rate of RS neurons at high RS firing rate by a constant value, but for a different reason. FS neurons, because of the depressing excitation from RS neurons, reach a maximal firing rate. In contrast, the firing rate of LTS neurons increases with *I*_R_, but the opening variable *s* of the depressing LTS-to-RS synapses saturates. Increasing *g*_FF_ reduces the maximal firing rate of the FS neurons (Equation 20) and their effects on RS neurons.

#### Dynamics of firing response to step inputs

**Figure 8:**
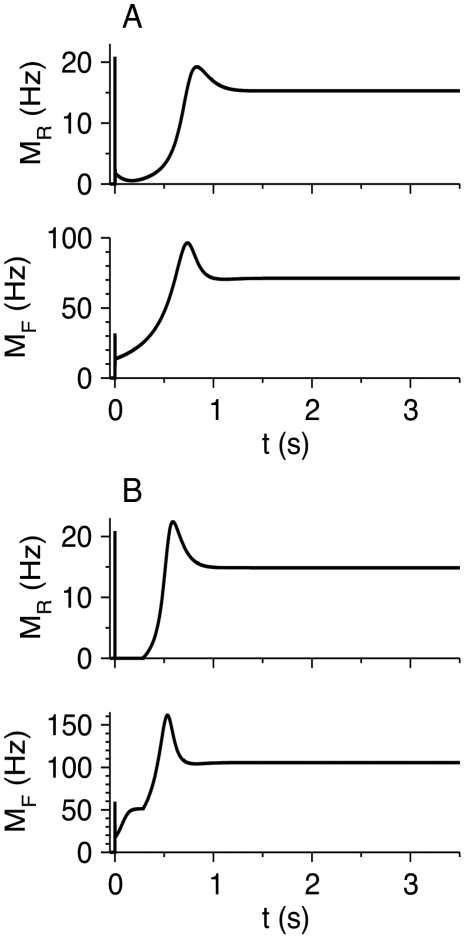
Response of the RS-FS network to step inputs *I*_R_Θ(*t*) and *I*_F_Θ(*t*) to the RS and FS neurons. Time courses of *M*_R_ (top panel) and *M*_F_ (bottom panel) are shown. Parameters: *g*_RR_ = 20, *g*_FR_ = 25, *g*_RF_ = 50, *g*_FF_ = 5, *I*_R_ = 0.29. (A) *I*_F_ = 0.35. (B) *I*_F_ = 0.45.

The temporal responses of *M*_R_ and *M*_F_ to step inputs *I*_R_ and *I*_F_ given at time *t* = 0 are presented in Figure 8A. The two neuronal populations respond to the steps with a brief, punctate response, after which the FS-to-RS inhibition rapidly reduces *M*_R_, and as a result *M*_F_ decreases as well. This component of the response resembles the brief experimentally observed response of RS neurons in vibrissa somatosensory cortex to whisker deflection because of feed-forward inhibition from FS neurons, known as the “window of excitability” [48], [49]. If *I*_F_ is large enough, *M*_R_ is reduced to zero (Figure 8B). Depression of the FS-to-RS synapses causes *M*_R_ to rebound. The rate *M*_R_ reaches a local maximum and then decreases somewhat towards its steady state value because *τ_r_*_,RR_<*τ_r_*_,RF_ (reference parameter set; Table 2). Without the RS-to-RS connections, the local maximum almost disappears; with strong RS-to-RS connections, fast oscillations, like those obtained in RS-LTS networks, may be generated (not shown).

### RS-LTS-FS Networks

**Figure 9:**
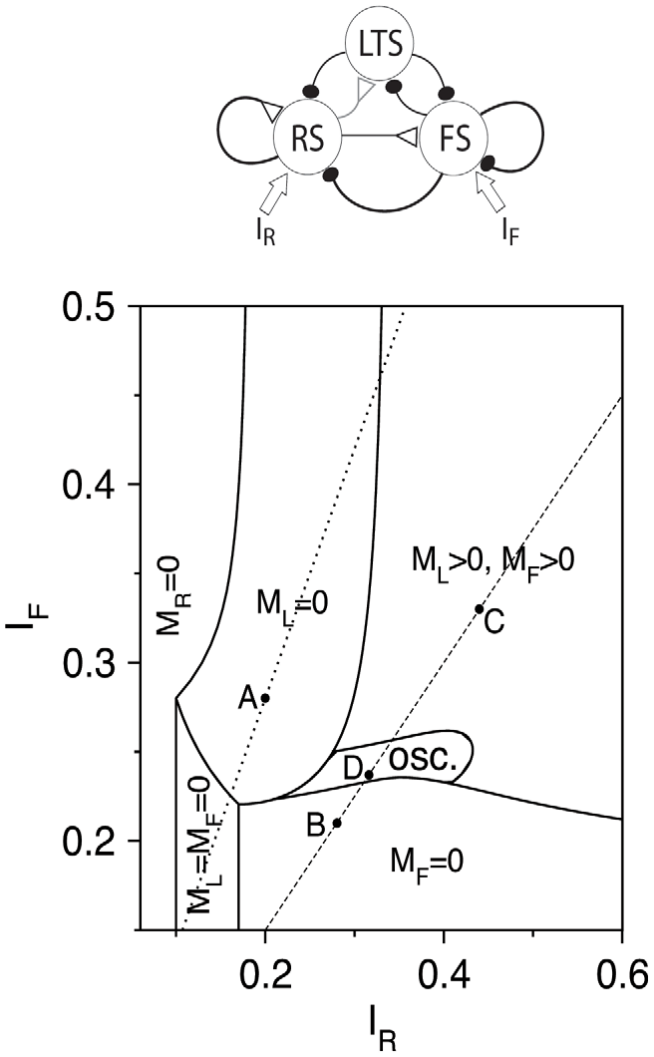
Phase diagram of the steady-state behavior of an RS-LTS-FS network (**Table 2**) in the *I*_R_−*I*_F_ plane. Regimes are defined according to the network state at large times. The network reaches a rest state with constant *M*_R_, *M*_L_, and *M*_F_ in all the regimes except of the oscillatory regime, denoted by “osc.”. In the regime denoted by “*M*_R_ = 0”, RS cells are quiescent; they are active in all other regimes. Those regimes are defined according to the activity of LTS and FS neurons. FS neurons are active and LTS neurons are quiescent in the regime denoted by “*M*_L_ = 0”, LTS neurons are active and FS neurons are quiescent in the regime denoted by “*M*_F_ = 0”, and both neuronal populations are active in the regime denoted by “*M*_L_>0, *M*_F_>0”. The dotted and dashed lines denote the ratios *I*_F_ = 1.4 *I*_R_ and *I*_F_ = 0.75 *I*_R_ respectively, for which calculations shown in Figure 10 are made. The solid circles labeled “A”–“D” denote values of *I*_F_ and *I*_R_ for Figure 11A–D.

Firing of excitatory neurons in cortex is controlled by inhibition from both LTS and FS interneurons, and we therefore characterize responses of the RS-LTS-FS network (Figure 1) to external input that may reach the RS and FS populations. We start by describing the steady-state response of the circuit with the reference parameter set (Table 2) to constant *I*_R_ and *I*_F_, as summarized in the phase diagram in Figure 9. The RS population is quiescent for small *I*_R_ (*M*_R_ = 0). It is active for larger *I*_R_ values, and the behavioral regimes in the phase diagram are denoted by the inhibitory population(s) that is (are) silent. Just above the RS firing threshold, and when *I*_F_ is small, both FS and LTS populations are quiescent (*M*_L_ = *M*_F_ = 0). For larger *I*_R_ values and for small *I*_F_, LTS neurons fire and FS neurons are quiescent (*M*_F_ = 0). For moderate values of *I*_R_ and large values of *I*_F_, LTS neurons are quiescent and FS neurons fire (*M*_L_ = 0). For large values of *I*_R_ and *I*_F_, both populations of interneurons are active (*M*_L_>0, *M*_F_>0). Between the last three regimes (*M*_F_ = 0, *M*_L_ = 0, and *M*_L_>0, *M*_F_>0), there is a state of slow oscillations, on the time scale of short-term synaptic plasticity (see below). This phase diagram remains qualitatively the same if the synaptic conductances are varied, except that fast oscillations, like those shown in Figure S4C, are observed for large *g*_RR_ (not shown).

**Figure 10:**
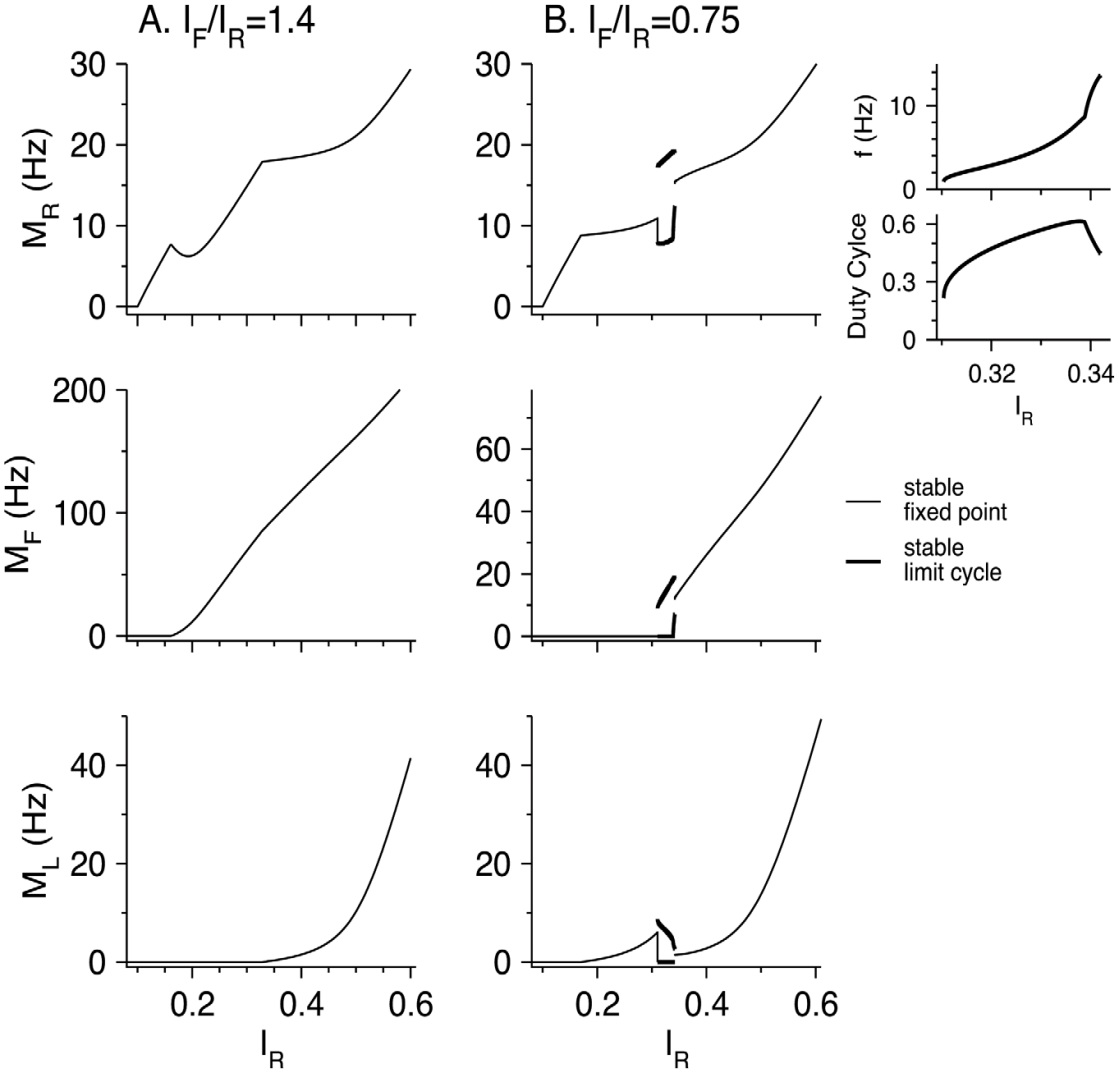
Steady-state response of the RS-LTS-FS network constant inputs to the RS and FS neurons with fixed *I*_F_/*I*_R_. Parameters are listed in Table 2. In the two left columns, values of *M*_R_ (top panels), *M*_F_ (middle panels) and *M*_L_ (bottom panels) are plotted as a function of *I*_R_. Thin solid lines: stable fixed points; thick solid lines: minimum and maximum of *M* on stable limit cycles (slow-oscillations states). (A) *I*_F_ = 1.4 *I*_R_ (dotted line in Figure 9). (B) *I*_F_ = 0.75 *I*_R_ (dashed line in Figure 9). The small panels on the right display the oscillation frequency *f* and the duty cycle of the oscillations (the ratio between the time interval during which RS neurons are in the more active state and the oscillation time period).

When thalamic input is varied, both *I*_R_ and *I*_F_ vary in a coordinated manner [15], [36]. Therefore, we examine how the steady state firing rates of the neuronal populations vary with *I*_R_ while keeping *I*_F_/*I*_R_ fixed. When *I*_F_/*I*_R_ = 1.4 (Figure 10A; denoted by a dotted line in Figure 9), the two inhibitory populations are quiescent just above the RS firing threshold. FS neurons start to fire for *I*_R_ = 0.16, and cause the RS firing rate to decrease. This decrease occurs because FS neurons receive independent input, *I*_F_, that increases with *I*_R_. As *I*_R_ continues to increase, *M*_R_ increases again because FS-to-RS synapses depress. For *I*_R_ = 0.33, LTS neurons start also to fire, and the RS gain decreases again before converging to *β*_R_ for very large *I*_R_. When *I*_F_/*I*_R_ = 0.75 (Figure 10B; dashed line in Figure 9) LTS neurons start to fire for *I*_R_ = 0.17 and reduce the RS gain, but do not make it negative because LTS neurons do not receive external input. Oscillations occur for 0.31<*I*_R_<0.34. For just above *I*_R_ = 0.34, FS and LTS neurons fire at steady state and reduce the RS gain. This gain increases with *I*_R_ and approaches *β*_R_ for large *I*_R_. Similarly, the gain of FS neurons approaches *β*_F_. Note that the value of *M*_L_ for *I*_R_ values just above the oscillatory regime (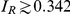) is smaller than its value just below this regime (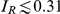), because FS neurons fire and inhibit LTS neurons.

At high rate, *M*_R_ increases linearly with *I*_R_ for all values of fixed *I*_F_/*I*_R_. This linear dependency is caused by LTS neurons only for low *I*_F_/*I*_R_ and by both LTS and FS neurons if *I*_F_/*I*_R_ is not low. Similar behavior is obtained for absence seizure thalamic input (not shown). The control of seizures by the two inhibitory populations is therefore qualitatively the same.

**Figure 11:**
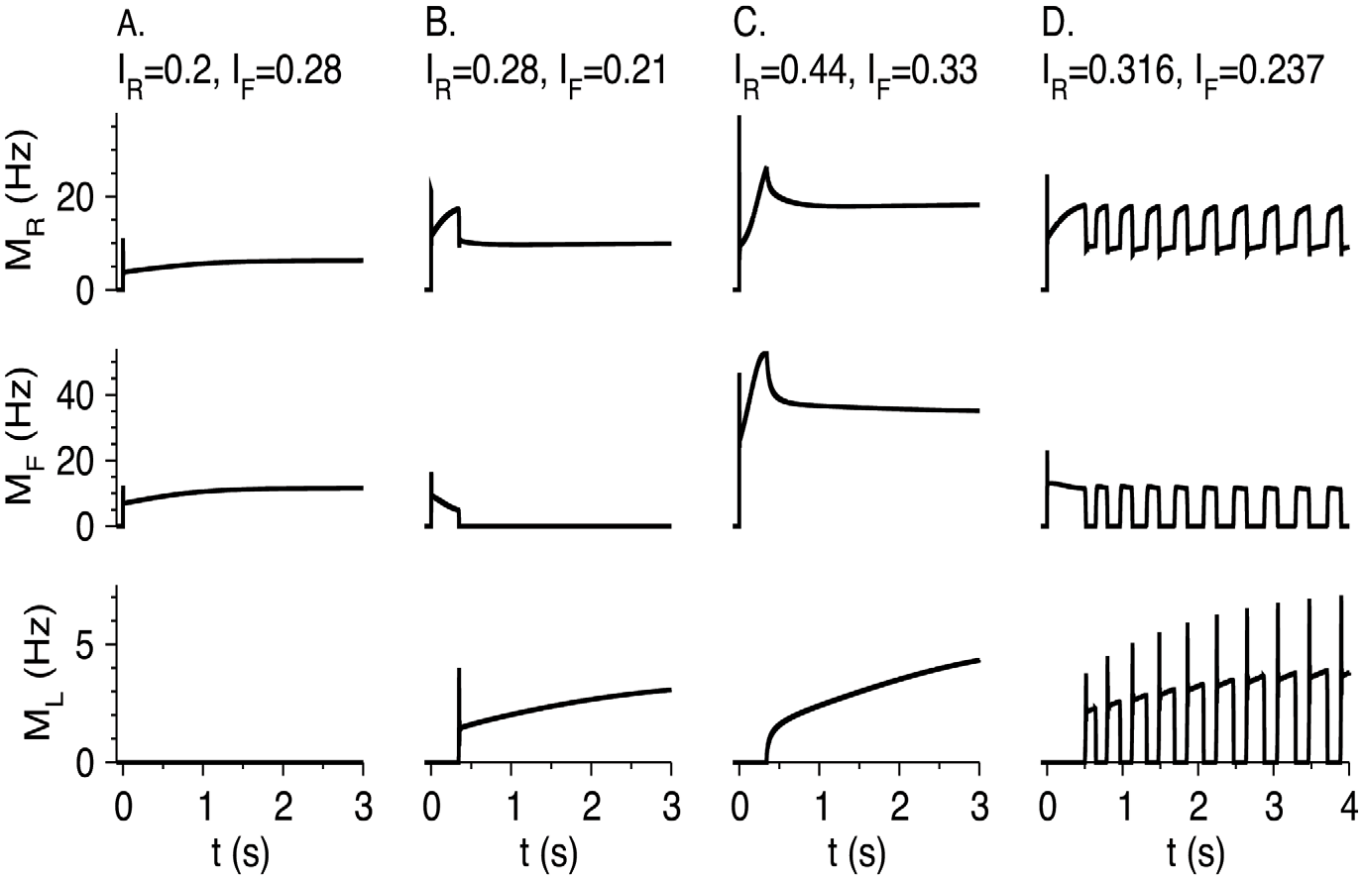
Response of the RS-LTS-FS network to step inputs *I*_R_Θ(*t*) and *I*_F_Θ(*t*) to the RS and FS neurons. Parameters are listed in Table 2. Time courses of *M*_R_ (top panels), *M*_F_ (middle panels) and *M*_L_ (middle panels) are plotted. (A) *I*_R_ = 0.2, *I*_F_ = 0.28. *M*_L_ = 0 for all times. (B) *I*_R_ = 0.28, *I*_F_ = 0.21. *M*_F_ = 0 for large *t*. (C) *I*_R_ = 0.44, *I*_F_ = 0.33. *M*_F_ and *M*_L_ are non-zero for large *t*. (D) *I*_R_ = 0.316, *I*_F_ = 0.237. The network oscillates at large *t*. RS neuron oscillate between a more active state and a less active state. FS neurons fire during the more active state of RS neurons, and LTS neurons fire during the less active state of RS neurons.

The dynamic response of three neuronal populations to step inputs *I*_R_ and *I*_F_ given at time *t* = 0 are presented in Figure 11A–D for four values of *I*_R_ and *I*_F_. In all cases, RS and FS neuronal populations respond to the step initiation by a brief firing during a “window of opportunity” before settling slowly to an attractor. In Figure 11A, representing the steady-state regime “*M*_L_ = 0”, those two populations increase slowly to their steady-state value after a rapidly-evolving initial response. In Figure 11B (“*M*_F_ = 0” in steady-state), RS and FS neurons are active during a time interval of a few tenths of seconds. Then, at about *t* = 0.35 s, LTS neurons start sharply to fire, whereas the activity of RS and FS neurons is reduced to non-zero and zero values respectively. In Figure 11C (“*M*_F_>0, *M*_L_>0” in steady-state), RS and FS are also active during an initial period of a few tenths of ms whereas the LTS neurons are silent. Here, however, the firing rate of LTS neurons increases continuously as they start to fire. The firing rates of the RS and FS neurons are reduced as a result of inhibition by LTS neurons, but both firing rates approach non-zero values at large times. The initial time courses of *M*_R_, *M*_F_, and *M*_L_ in Figure 11D (oscillations) are similar to those in Figure 11B. At longer times, however, the time courses converge to an oscillatory state. Interestingly, the amplitude of LTS oscillations develops more gradually towards its steady-state value than the amplitudes of RS and FS oscillations. During the oscillatory state, RS neurons oscillate between a more-active phase and a less-active phase, where the firing rate in both phases is larger than zero. FS neurons fire episodes of spikes, represented by positive *M*_F_, when the RS neuronal population is in its more-active phase. They are quiescent when the RS neurons are less active. LTS neurons oscillate in opposite phase: they fire when RS neurons are in the less-active phase, and are quiescent otherwise. The oscillation frequency is on the order of a few Hz, corresponding to the time scale of short-term synaptic plasticity, and it increases as *I*_R_, and therefore *I*_F_, increases (Figure 10B, top-right). The duty cycle of the more-active state is defined as the time that the RS population spends in that state (and the FS neurons are active) divided by the time period. This ratio varies from 0.2 to about 0.6, and it first increases and then decreases with *I*_R_ (Figure 10B, bottom-right).

### Mechanism of Slow Oscillations in RS-LTS-FS Circuits

**Figure 12:**
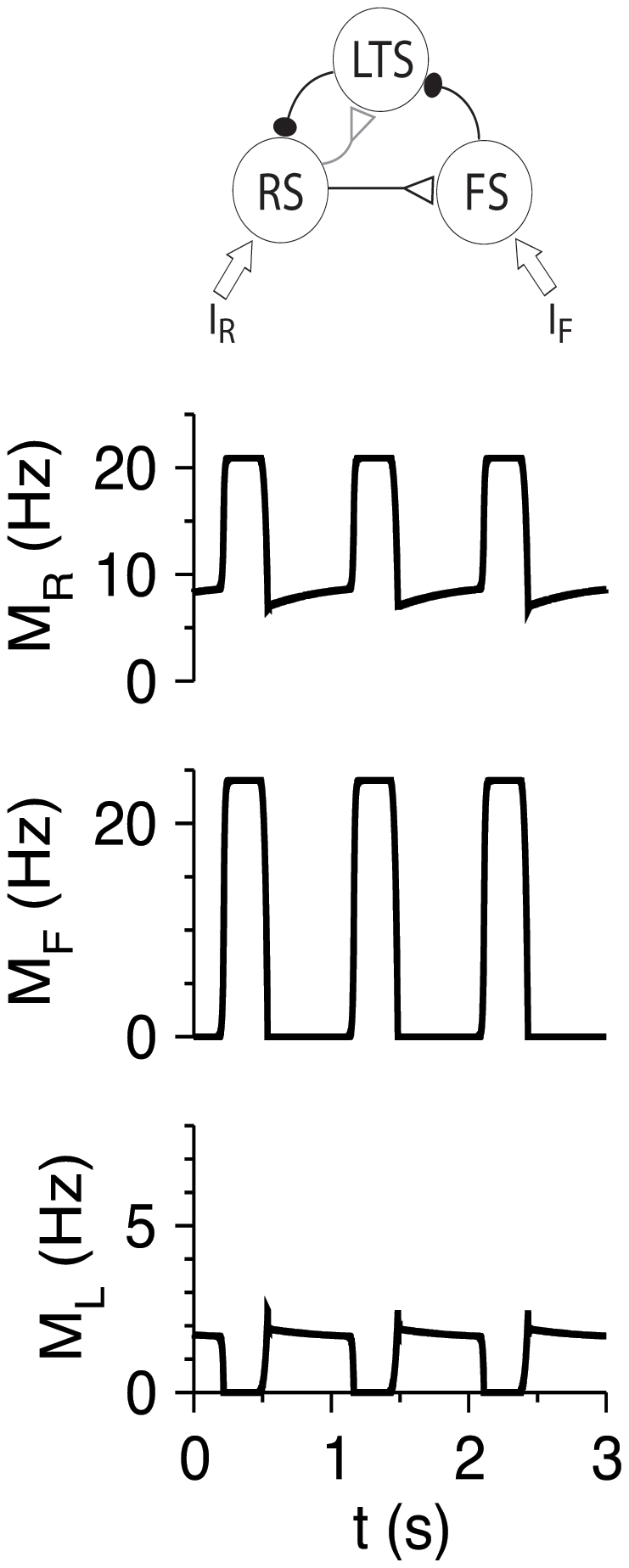
Oscillatory response of the reduced RS-LTS-FS network to constant inputs. Time courses of *M*_R_ (top panels), *M*_F_ (middle panels) and *M*_L_ (middle panels) are plotted during the oscillatory state (limit cycle). Parameters that are different than those listed in Table 2 are: *g*_LR_ = 7.5, *g*_RR_ = 0, *g*_FR_ = 9.3, *g*_LF_ = 8, *g*_RR_ = *g*_RF_ = *g*_FL_ = *g*_FF_ = 0, *τ_r_* = 0 for all the synapses. Additional parameters: *I*_R_ = 0.29, *I*_F_ = 0.232.

We find that a slow oscillation state appears in our model only when it includes the two neuronal populations, whereas models of RS-LTS networks and RS-FS networks exhibit either rest states or, in restricted values of *g*_RR_, fast oscillations. What is the dynamical mechanism that leads to the slow oscillations state? Such states are often studied using fast-slow analysis [50], [51], [52], [53], [54], [55]. In our case, equations 1–6 for the RS-LTS-FS network (Figure 1) include 8 slow variables, and it is practically impossible to analyze them. Fortunately, we find that slow oscillations still prevail in a reduced RS-LTS-FS circuit with only RS-to-LTS, LTS-to-RS, RS-to-FS and FS-to-LTS synaptic connections and without short-term plasticity properties of the depressing synapses, *i.e. τ_r_* = 0 for all the synaptic connections (Figure 12). Facilitation of the RS-to-LTS synapses is, however, necessary to maintain the oscillations.

**Figure 13:**
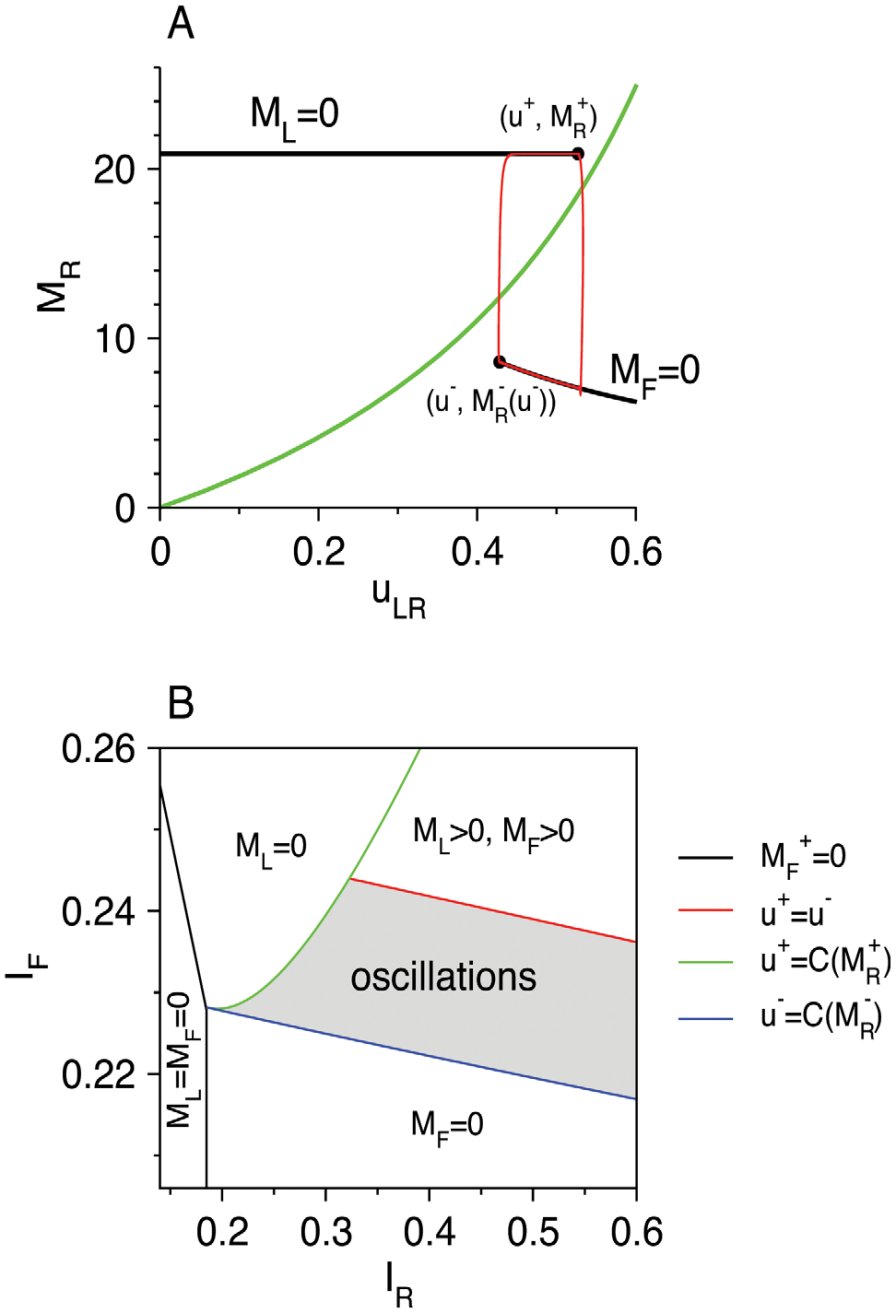
Fast-slow analysis of network oscillations: analysis of the reduced model. Synaptic parameters are as in Figure 12. (A) The bifurcation diagram of the fast subsystem is presented by plotting *M*_R_ as a function of the parameter *u*_LR_. Solid black lines denote branches of stable fixed points; *M*_L_ = 0 on the upper line and *M*_F_ = 0 on the lower line. The points 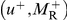 and 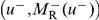 (Equations 31, 32) are denoted by black solid circles. The green line denotes the slow nullcline of Equation 30, *u*_LR_ = *CM*_R_/(1+*CM*_R_). The red line denotes the projection of the limit cycle of the full dynamical system (Equations 1, 3–6) on the *M*_R_–*u*_LR_ plane. Parameters: *I*_R_ = 0.29, *I*_F_ = 0.232. (B) Phase diagram of the RS-LTS-FS network in the *I*_R_−*I*_F_ plane. The network exhibits slow network oscillations in the grey area. Outside of this regime, the network reaches a steady state with constant *M*_R_, *M*_L_, and *M*_F_. The LTS and FS populations are quiescent to the left of the black line. To the right of the black line and below the blue line, *M*_F_ = 0 and *M*_L_>0. To the right of the black line and to the left and above the green line, *M*_L_ = 0 and *M*_F_>0. To the right of the green line and above the red line, *M*_L_>0 and *M*_F_>0.

The reduced system has only one slow variable, *u*_LR_, and all the other variables are much faster. We use the technique of fast-slow analysis (See “Fast-slow analysis of slow network oscillations” in Methods) to define the mechanism of slow oscillations. We find that in order for the slow oscillations to emerge, the fast subsystem that includes all the variables except *u*_LR_ should be bistable (Figure 13A). In one stable state, denoted “more active”, LTS neurons are silent and RS and FS neurons are active. In the second state, denoted “less active”, LTS neurons are active, RS neurons are active, but less than in the more active state, and FS neurons are silent. The dynamics of the full system switch rapidly back and forth between these two states. This explains the pattern of activation seen in the reduced RS-LTS-FS system (Figure 12) as well as in the full system (Figure 11D). Bistability ceases to exist if *I*_F_ and *I*_R_ are large enough, and in this case the system reaches a steady state in which both *M*_L_ and *M*_F_ are non-zero (Figure 13).

Another condition needed to obtain oscillations is that the fixed point of the full dynamical system is not stable. This condition is broken if *I*_F_ is too small, and then the system converges to a steady state where *M*_F_ = 0 and *M*_L_>0 (see “Borders of the regime of slow network oscillations in the phase diagram” in Methods). It is also broken if *I*_R_ and *I*_F_ are small and large enough respectively. In this case, the system converges to a steady state where *M*_F_>0 and *M*_L_ = 0. Qualitatively, this behavior is also shown by the original RS-LTS-FS network (Figure 9). Analysis of the reduced system also reveals that the oscillatory regime extends over a limited range of *I*_F_ (Figure 13B and Methods), as also found for the full model (Figure 9). The oscillatory regime of the reduced model extends over a larger *I*_R_ range. This range is more limited in the case of the full model (Figure 9), probably because of the effects of synaptic depression.

## Discussion

### Summary of Results

Because of the facilitatory nature of RS-to-LTS connections, it was hypothesized that these neurons prevent overactivation and seizures by reducing cortical activity mostly at high rates [9], [18], [19]. It was also suggested that they do so by decreasing the gain of pyramidal cell output [23]. However, we find that the dynamical picture is different due to the LTS-to-RS synaptic depression. At high firing rates, LTS neurons do not change the RS gain at all, and reduce the firing rates of RS neurons by a constant value, independent of the input *I*_R_. Importantly, LTS neurons do reduce RS gain at modest firing rates, just above the LTS firing threshold, where LTS-to-RS depression is weak [38]. LTS neurons therefore have a divisive effect on the RS firing at modest rates and a subtractive effect at high rates. Their effect at high rates is therefore limited, because a divisive effect is more potent than a subtractive one during gradual elevations in cortical activity as it increases with the elevation of firing rates. Responses to absence-seizure-like inputs are qualitatively similar to the response to step inputs. Although RS-to-LTS synapses facilitate and RS-to-FS synapses depress, the two inhibitory populations reduce the firing rates of RS neurons in a similar manner at high rates. In response to input step currents, RS cells in all three networks (RS-LTS, RS-FS and RS-LTS-FS) respond with a brief firing epoch followed by reduced firing (and even quiescence) and then rebound to larger firing rates. This initial firing epoch terminates faster for FS neurons than for LTS neurons.

An RS-LTS-FS network usually reaches a steady state with FS neurons quiescent for small *I*_F_, LTS neurons quiescent for small *I*_R_, and both populations active for large *I*_R_ and *I*_F_. Between these behavioral regimes, there is a relatively narrow regime of slow (few Hz) oscillations. These oscillations are induced by the slow facilitation variable of the RS-to-LTS synapses that transfers the system alternately between two bistable states of the fast dynamics. During these oscillations, RS neurons switch from a more-active to a less active state alternately, whereas LTS and FS neurons switch alternately from an active state to a silent state. In general, FS neurons tend to follow the spiking of RS neurons closely, whereas LTS neurons follow it with delays.

Inhibitory neurons can reduce the response of their targets by either shifting the target's response curve (a subtractive effect) or by reducing its gain (a divisive effect). A simple biophysical model without synaptic depression predicts constant inhibition (*i.e.*, independent of the activity of the target) and causes a subtractive effect [29], [56]; this result is confirmed experimentally [57]. If the activity of the inhibitory neurons is caused by the firing pattern of the excitatory target population, the effect is divisive (Figure 2B, blue curve), whether the excitatory-to-inhibitory synapses facilitate or not. We show, using a rate model, that synaptic depression in the inhibitory-to-excitatory synapses exhibits similar divisive behavior at low rates, where depression effects are small. At high rates, depression causes the effect to be subtractive because the efficacy of the depressed inhibitory synapse scales as one divided by the firing frequency of its presynaptic inhibitory neuron.

### RS-LTS vs. RS-FS Networks

RS-LTS networks are different from RS-FS circuits primarily because of the facilitating nature of RS-to-LTS synapses versus the depressing nature of the RS-to-FS synapses, and because FS neurons receive strong external input [9]. As a result, tested independently of one another, the LTS and FS inhibitory populations have distinctly different effects on the input-output properties of cortical circuits that are demonstrated at steady states and low firing rates. LTS neurons do not affect the minimal input level *I*_R_ above which RS neurons fire. Just above the LTS firing threshold *I*_R.LTS,th_, LTS neurons affect the RS gain most strongly, but reduce *M*_R_ less strongly than at high rates. The value *I*_R.LTS,th_ decreases with *I*_L_ if LTS neurons receive their own thalamic input. In contrast, FS neurons, which receive substantial external input, increase the current threshold *I*_R_ for RS firing, do not considerably affect the RS gain, and reduce *M*_R_ effectively starting from just above this threshold. The effect of FS neurons on RS firing is therefore always subtractive. At high firing rates, both the LTS and the FS neuronal populations affect the RS firing properties in a similar manner by reducing the firing rate of RS neurons by a constant value. The reasons for this behavior, however, are different: the LTS input to RS neurons reaches a constant value at high rates because of the LTS-to-RS synaptic depression (Figure 2), whereas FS input to RS neurons is limited by the saturation of the firing rate of FS neurons themselves (Figure 7).

In response to step input currents, LTS neurons respond with a delay just above *I*_R,LTS,th_ (Figure 3). After the delay, LTS neurons decrease the activity of RS neurons to a minimal value, after which *M*_R_ rebounds. FS neurons reduce the activity of RS neurons much more rapidly after a stimulus onset, leaving only a brief “window of opportunity” for RS initial firing (Figure 8). The RS activity then decreases to low (even zero) values before rebounding to its steady-state values. Interestingly, the temporal profiles of *M*_R_ in the RS-LTS network with large *I*_R_ and RS-FS networks are similar (Figures 3A and 8), except that the initial decay of *M*_R_ in the RS-LTS network is more gradual. The temporal profiles of the activity of the two inhibitory neurons in these networks are, however, different: FS neurons, but not LTS neurons, respond with brief initial activity to the step input activity. Strong RS-to-RS connections may induce fast oscillations in both circuits (Figures S3,S4) [28], [58].

In general, FS cells tend to track spiking of the RS cells much more closely than the LTS cells do. This behavior is seen by comparing RS-FS and RS-LTS circuits (Figures 3, 8) as well as in RS-LTS-FS circuits (Figures 11, 12). FS and LTS neurons behave dynamically quite differently from one another.

### Response of RS-LTS-FS Circuits to External Inputs

At steady state, cortical networks with active RS neurons show four types of resting states in which: only LTS neurons are active, only FS neurons are active, both interneuron populations are active, or neither is active (Figure 9). The oscillatory regime is located near the intersection of all these states. Despite the fact that it is narrower than the other states, analysis of its existence determines the structure of the other states. If the fast subsystem of variables ceases to be bistable as a parameter is varied, a state with active LTS and FS neurons is obtained. If the fast subsystem is bistable and a rest state of the full subsystem occurs on a branch with FS (respectively LTS) neurons quiescent, a state with a quiescent FS (respectively LTS) population is obtained. We show this theoretically in a reduced circuit (Figure 13) and numerically in the full circuit (Figure 9). In the parameter regimes when LTS neurons are active in the steady state, the initial response to step currents is similar to that in the oscillatory regime (Figure 11). RS and FS neurons are active in the initial several tenths of one second while LTS neurons are silent. Then, LTS neurons start to fire and reduce the firing rate of FS neurons.

When thalamic input varies, it is expected that *I*_R_ and *I*_F_ will vary proportionally [36]. Increasing the input can therefore cause non-monotonic variation of the firing rate of one of the neuronal populations, with or without passing through the oscillatory regime (Figure 10). In RS-LTS-FS circuits, as in circuits with one population of interneurons only, the gain of RS and FS neurons at high rates is not affected by the circuit.

### Slow and Fast Oscillations in Cortical Circuits

Our cortical circuit model exhibits two types of cortical oscillations. Large *g*_RR_ may generate fast oscillations, as was shown in previous models of cortical circuits [28], [59]. One type of inhibitory interneuron, either LTS or FS, is sufficient for the generation of fast oscillations, together with large (but not extremely large) values of *g*_RR_. The oscillation frequency is on the scale of 1/*τ_s_*, about tens of Hz. Excitatory and inhibitory neurons fire nearly in phase (Figure S4C), and there is a substantial time interval in each period during which both neuronal populations are quiescent.

In this study, we discovered a novel type of oscillation in cortical networks that depends on RS-to-LTS synaptic facilitation and on external input to the FS neurons, and can occur without any RS-to-RS recurrent excitation. These oscillations have several characteristics. Both LTS and FS populations are necessary for generating them. The oscillation frequency, ∼1–10 Hz, is on the time scale of 1/*τ_f_*_,LR_, the facilitation recovery time constant. RS neurons oscillate between more-active and less-active states, both with positive firing rates. FS and LTS neurons fire in phase and in anti-phase with the RS more-active state, respectively. Hence, in contrast to the fast oscillations, at least one population of neurons is active at every time point.

Slow cortical oscillations have been observed during sleep, anesthesia and quiet wakefulness *in vivo* [60], [61], [62], [63], *in vitro* [64] and in computational models [26], [65]. During these oscillations, the neurons in the network switch from an active “up” state to a silent “down” state and back. The oscillations we observe in the RS-LTS-FS model are different from those oscillations because the RS neurons during the less-active state are not silent, and because the LTS neurons fire during the less-active state. Cortical oscillations with a frequency on the order of 1 Hz, during which the network is not completely silent during the less-active state, have also been observed [66], [67], and spontaneous activity was observed during which neurons fired in episodes with similar frequencies [68]. Using future recordings from LTS and FS neurons *in vivo* [17], or using optogenetics techniques to activate RS or FS populations selectively [15], it will be possible to determine whether LTS neurons are active during the less-active state of the RS populations, as the theory predicts.

Interestingly, the frequency range (∼1–10 Hz) of the slow oscillation observed in our RS-FS-LTS model overlaps with that of absence seizures and both the tonic and clonic phases of tonic-clonic seizures [44]. While other mechanisms may contribute to these seizure components (e.g. rhythmic thalamic input in absence seizures), the oscillatory pattern observed in our model could conceivably perpetuate or reinforce such pathological conditions. It remains to be seen whether FS and LTS cells alternate their firing during these conditions, as suggested by our results.

### Comments on Our Theoretical Approach

Each neuronal population is represented in our model by its firing rate. Rate models can describe the properties of large networks of neurons represented by conductance-based schemes provided that the level of synchrony in the network is small, and the input is stationary or slowly modulating in time [29]. The level of synchrony in cortical networks, especially in awake animals, is often small [69], [70]. Therefore, our rate model is expected to describe the dynamics of cortical networks that receive stationary input reasonably well in comparison to more complicated models of spiking neurons. In addition, we examine the response of networks to step or absence-seizure-like inputs. In such cases, the outcome of rate models should be regarded as a qualitative estimation of the full dynamics. In particular, neurons often show sharply transient responses to step inputs when FS neurons play a major role in the dynamics. Using rate models we can claim that such a response occurs, but cannot determine its properties on time scales of milliseconds.

Our basic form of the model does not include spike-frequency adaptation and firing-rate saturation. Adaptation does not change the steady-state response of the circuits. Dynamically, with the slope of the *f*-*I* curve scaled to be equal with and without adaptation (Equation 19), a model with adaptation exhibits a stronger initial response to step inputs, whereas its subsequent long-term response is similar to that of the model without adaptation (Figure 5). Saturation reduces the activity at high rates but does not change the qualitative effects of LTS and FS inhibition on the cortical circuit.

LTS neurons project mostly to distal dendrites of pyramidal neurons [4], [5], but their inhibitory effects are clearly observed in the soma [8], [18], [19], [20]. Such effects can be described by the rate model presented here, which is based on linear summation of inhibitory PSPs in the soma [29]. Developing more elaborate rate models, that can account for spatial properties of neurons and describe LTS effects on local dendritic computation [71], remains a challenge.

We use the fast-slow analysis to determine the conditions for obtaining slow oscillations. This analysis is often used when one or several time constants in the system are much larger than the other time constants [51], [52]. We apply the method to our reduced circuit (Figure 13) with no synaptic depression, by assuming that both *τ_f_*_,LR_ is large and *U*_LR_ is small. These approximations yield good fits of the predictions of the fast-slow analysis (Figure 13) to the full dynamics of the reduced system, computed using numerical simulations (Figure 12). The phase diagram (Figure 13B) of the reduced circuit is qualitatively similar to that of the full circuit (Figure 9) and displays the same behavioral regimes, but the locations of the borders between the regimes in the phase diagrams of the two circuits are quantitatively different.

### Comparison with Previous Theoretical Work

Most models of the response of cortical circuits (e.g., [28], [58], [59], [72]) to input do not consider short-term synaptic plasticity. Like our model, these models can show fast oscillations as a result of interactions between excitatory and inhibitory neurons. The contribution of LTS neurons was shown to shape the response of cortical circuits to periodic inputs [73] in a model with short-term synaptic plasticity of excitatory synapses but without considering depression of inhibitory synapses.

While our model may exhibit slow oscillations with facilitation of the RS-to-LTS synapses and depression of all other synaptic connections (Figure 11D), depression is not necessary for obtaining oscillations (Figure 12). In contrast, depression is essential for various slow oscillations in other models of cortical networks [24], [25], [26]. Facilitation of the excitatory-to-inhibitory synapses generates slow oscillations in a rate model of cortical circuits composed of excitatory and inhibitory populations [27]. Inhibitory neurons in that model receive external input and are mutually coupled by inhibitory synapses. In our model, inhibitory LTS neurons receive facilitating input from excitatory RS neurons, but do not receive external input and are not mutually coupled, according to circuit properties discovered experimentally [9]. Excitatory and inhibitory populations in the model of Melamed *et al.* [27] fire during the same phase interval during the cycle, whereas LTS and RS neurons in our model fire in anti-phase. Another difference is that the firing rate of excitatory neurons during the “down” state in the Melamed *et al.* model is zero, whereas the firing rate of the RS neurons in our model during the less-active state is positive.

### Functional Significance

Roles of specific types of interneurons in diseases such as epilepsy [21], [22] and schizophrenia [74] have been suggested. By analyzing a rate model of cortical circuits with Tsodyks-Markram kinetics for short-term synaptic plasticity, we observe that in response to high input *I*_R_, the LTS population reduces the firing rate of the RS neurons by a constant factor, independent of *I*_R_. We demonstrate this behavior specifically for a model with absence-seizure-like input. This implies that LTS neurons can help to prevent seizures in cortex, but the role of LTS cells in this task is qualitatively as limited as that of FS neurons. Indeed, selective damage to the LTS neurons (for which there is evidence in experimental seizure models and human cortex) may be compensated by FS neurons or by other types of inhibitory interneurons such as neurogliaform cells [23]. Our results are consistent with experimental results showing that the death of LTS interneurons does not initiate hyperexcitability in a neonatal rat model of human polymicrogyria, which is often characterized by severe seizures [75].

Whereas most of our calculations are carried out for constant or step stimuli, our results are applicable also for pulsatile thalamic input (Figure 6), at least above 3 Hz. Increasing the frequency will make the approximations of the model even more accurate. During whisking, cortical circuits receive periodic thalamic input at frequencies of about 10 Hz [76]. Similarly, visual thalamic input to cortex is often described as Poisson firing, with firing rates of about 20 Hz [77]. Since the time constants of synaptic depression and facilitation are much longer, the slow dynamics will average over the spiking process and will depend on the underlying firing rate, similar to the response to constant or slowly-varying stimuli. Therefore, our finding that LTS neurons have a strong impact on the response to modest thalamic input, and not just during high frequency activity, are valid also for the cortical response to somatosensory and visual stimuli.

Our conclusion is an outcome of the depression kinetics of the Tsodyks-Markram model, where the total synaptic input reaches a saturating value as the presynaptic firing rate, *M*, increases. Saturation occurs because the additional postsynaptic conductance in response to one additional presynaptic spike scales as 1/*M* [38]. In various other types of depressing synapses characterized experimentally and using models, the response to an additional spike is larger than expected by the Tsodyks-Markram model, probably because the recovery from depression is faster at high presynaptic rates [78], [79], [80]. One reason we use the Tsodyks-Markram model in this work is that Silberberg and Markram fit their data for RS-to-LTS and LTS-to-RS synapses to it [18]. The theoretical results, however, suggest that the kinetics of these synapses in a broad frequency range should be measured in a more detailed manner.

In this work, we observe that LTS neurons affect the gain of RS neurons at rates on the order of 10 Hz and less. These rates are comparable with the rates of LTS neurons measured in vitro during a variety of diverse activating conditions [7], such as group I metabotropic glutamate or muscarinic cholinergic receptor agonists. Therefore, LTS neurons can affect cortical dynamics even if cortical neurons do not fire at high rates.

## Methods

### Model Parameters

The parameters of the neuronal populations are provided in Table 1. They were determined based on the experimental observations of Fanselow *et al.* [7]. The parameters of the synaptic connections are written in Table 2. These parameters are used in all calculations unless otherwise stated. The parameters determining the short-term synaptic plasticity properties of LTS-to-RS and RS-to-LTS synapses are taken from Silberberg and Markram [18] who carried out experiments in layer 5. This layer is most active in the initiation [81] and horizontal propagation of epileptiform [82] and normal [64] activity in the neocortex. Short-term plasticity parameters for the RS-to-RS synapses are taken from [83], and those for the FS-to-RS and RS-to-FS are taken from [47], [84]. We are not aware of any systematic research on the short-term synaptic plasticity properties of FS-to-FS, LTS-to-FS and FS-to-LTS connections, except that these synapses depress [85]. Therefore, we use the generic values *τ_r_* = 400 ms and *U* = 0.3. To simplify the analysis, we assume that *τ_r_* = 0 if *τ_r_*<<*τ_f_* and *τ_f_* = 0 if *τ_f_*<<*τ_r_*. The constant *τ_s_* is taken for AMPA and fast GABA_A_ excitation, and it is larger for the LTS-to-RS synapses than for the FS-to-RS synapses [9], [18].

### Threshold for LTS Firing for *g*_RR_ = 0

LTS neurons fire if *s*_LR_>θ_L_/*g*_LR_ (Equation 14). Using Equation 11, we find that LTS neurons fire for *M*_R_>*M*_R,th_, where
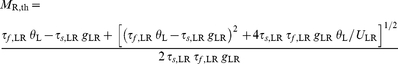
(22)The rate *M*_R,th_ is obtained for the LTS firing threshold *I*_R_ = *I*_R,th_, where
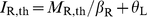
(23)From Equation 12, *s*_RL_≈*U*_RL_ *τ_s_*_,RL_ *M*_L_ for *M*_L_<<1. Using Equations 13, 14 we find that just above *I*_R,th_,
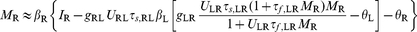
(24)Differentiating both sides of Equation 24 with respect to 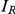, we obtain that the RS gain, *d*M_R_/d*I*_R_, is
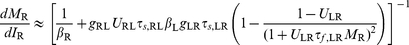
(25)


### Delay of LTS Firing in Response to Step Input

During the delay period, *M*_R_ = *β*_R_(*I*_R_−*θ*_R_). From Equation 8,
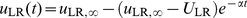
(26)where
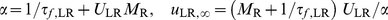
(27)Since *τ_f_*_,LR_>>*τ_s_*_,LR_, *s*_LR_ reaches a quasi-steady-state value, *s*_LR_ = *τ_s_*_,LR_ *u*_LR_ *M*_R_ (Equation 7). LTS neurons start to fire when *g*_LR_ *s*_LR_ = *θ*_L_, *i.e.*, when *u*_LR_ reaches the value *u*_LR,th_ = *θ*_L_/(*g*_LR_ *τ_s_*_,LR_ *M*_R_). From Equation 26, the delay time is
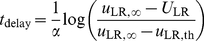
(28)


### Fast-Slow Analysis of Slow Network Oscillations

The reduced RS-LTS-FS dynamical system (Figure 12) has five dynamical variables. The four variables *s*_LR_, *s*_RL_, *s*_FR_, *s*_LF_, are fast, with *τ_s_*, on the order of a few ms (Table 2). The fifth equation (Equation 3), describing the facilitation process of the RS-to-LTS synapses, is
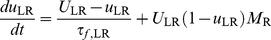
(29)We use the method of fast-slow analysis to describe the dynamics of the system for both large *τ_f_*_,LR_ and small *U*_LR_. Formally, we define *C*≡*U*_LR_ *τ_f_*_,LR_ and study the system in the limit *τ_f_*_,LR_→∞ and constant *C*. This approximation is expected to be justified for the RS-to-LTS synapses because *τ_f_*_,LR_, 670 ms, is two order of magnitude larger than the *τ_s_*'s, and *U*_LR_, 0.09, is an order of magnitude smaller than 1. Using the definition of *C* and neglecting a term on the order of 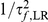, Equation 29 becomes
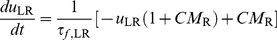
(30)


The full dynamical system describing the network can be separated into a fast subsystem, composed of the four equations for the variables *s*, and a slow subsystem, that includes the variable *u*_LR_. The first step in this method is to study how the attractors of the dynamics of the fast subsystem depend on the value of *u*_LR_, taken as a time-independent parameter. In a second step, one derives the dynamics of the full system taking into account the slow variations of *u*_LR_ (Equation 30).

The bifurcation diagram of the fast subsystem as a function of *u*_LR_ for the parameter set of Figure 12 is plotted in Figure 13A. The subsystem can settle into stable fixed points that belong to one of two branches. The upper branch is characterized by *M*_L_ = 0, *M*_F_>0, and a high value of *M*_R_, denoted by 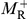, that does not depend on *u*_LR_. This branch exists for small *u*_LR_ values and disappears for *u*_LR_ = *u* ^+^ at a saddle-node bifurcation [50], where it coalesces with an unstable branch (not shown). The lower branch is characterized by *M*_F_ = 0, *M*_L_>0 and a low value of *M*_R_, denoted by 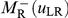, that depends on *u*_LR_. This branch exists for large *u*_LR_ values and disappears for *u*_LR_ = *u* ^−^ at a second saddle-node bifurcation.

The slow nullcline of Equation 30, characterized by *u*_LR_ = *CM*_R_/(1+*CM*_R_), does not intersect with either of the stable branches. Therefore, the full system does not have any stable fixed point. Instead, it exhibits relaxation-oscillation dynamics [50]. The system converges rapidly to one of the two stable branches of the fast subsystem. If it converges to the upper branch, it will then progress slowly to the “knee” at *u*_LR_ = *u* ^+^ and then will move rapidly to the lower branch. On that branch, the system progresses slowly to *u*_LR_ = *u* ^−^ and then moves rapidly to the upper branch, completing the oscillatory cycle. The trajectory of the full dynamical system with the reference parameter set that is overlaid on the bifurcation diagram in Figure 13A fits this bifurcation picture very well. This fit shows that analysis in the limit *τ_f_*_,LR_→∞ and constant *C* describes well the dynamics with biologically realistic parameters.

### Borders of the Regime of Slow Network Oscillations in the Phase Diagram

The fast-slow analysis yields three conditions that together are both necessary and sufficient for the generation of slow oscillations:

1. To enable bistability, *u* ^+^>*u* ^−^.
2. The upper branch should not intersect with the slow nullcline,
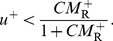
(31)
3. The lower branch should not intersect with the slow nullcline,
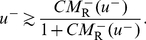
(32)

We calculate *u* ^+^ (resp. *u* ^−^), the value of *u*_LR_ above (resp. below) where the upper (resp. lower) branch of the fixed points of the fast subsystem no longer exists (Figure 13A). We define 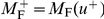, 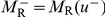 and 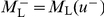. From Equation 1, at a steady state of the fast subsystem,
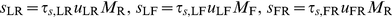
(33)LTS neurons fire above *u* ^+^. According to Equation 5, at the onset of LTS firing (*M*_L_ = 0^+^),
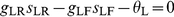
(34)Substituting Equation 33 in Equation 34, we obtain for *u*_LR_ = *u* ^+^,
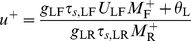
(35)Using Equations 4, 6 and 33, and because FS neurons are active and LTS neurons are silent on the upper branch, we obtain
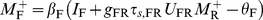
(36)

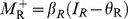
(37)To calculate *u* ^−^, the value of *u*_LR_ below which the lower branch of the fixed points of the fast subsystem no longer exists (Figure 13A), we note that FS neurons fire below this value. According to Equation 6, at the onset of FS firing (*M*_F_ = 0^+^),
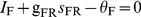
(38)Substituting Equation 33 in Equation 38, we obtain for *u*_LR_ = *u* ^−^,
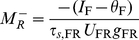
(39)From Equations 4,5 and 33, and because LTS neurons are active and FS neurons are silent on the upper branch, we obtain
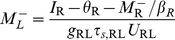
(40)

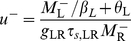
(41)The parameter regime that fulfills the three conditions written above (*u* ^+^>*u* ^−^ and Equations 31–32, computed using Equations 35–37, 39–41) is denoted in a phase diagram in the *I*_R_–*I*_F_ plane (Figure 13B). Slow oscillations are observed for levels of *I*_R_ that are not too small and levels of *I*_F_ within a certain narrow range. This range is always below *θ*_F_, such that excitation from RS neuron is needed to induce firing in the FS neurons. Above a certain value of *I*_R_ (0.32 in Figure 13B), this *I*_F_ range has an (almost) constant width, and its borders decrease (almost) linearly with *I*_R_. Outside of the oscillatory regime, the network reaches a steady state. For large *I*_R_ and small *I*_F_, *M*_F_ = 0 and *M*_L_>0. For large *I*_R_ and *I*_F_, *M*_F_>0 and *M*_L_>0. For large *I*_R_ and medium values of *I*_F_, *M*_F_>0 and *M*_L_ = 0. Finally, for small *I*_R_ and *I*_F_, the two inhibitory neuronal populations are quiescent.

### Numerical Methods

Simulations were performed using the fourth-order Runge-Kutta method with a time step of 0.02 ms implemented as a C program or within the software package XPPAUT [86], which was used also for computing the bifurcations of fixed points in the diagram in Figures S4A.

## Supporting Information

Figure S1 **Effects of firing rate saturation.** *M*_R_-*I*_R_ curves (top panel) and *M*_L_-*I*_R_ curves (bottom panel) are plotted for *g*_RL_ = 0 (black) and 35 (red). Solid line: *M_i_* values are calculated according to Equation S1; dotted line: *M_i_* values are calculated according to Equations 4–6. Additional parameters are *g*_RL_ = 35, *g*_LR_ = 7.5, *g*_RR_ = 0.
(EPS)

Figure S2 **Steady-state response of the RS-LTS network with RS-to-RS synaptic connections to constant inputs to the RS neurons.** Additional parameters are *g*_RL_ = 35, *g*_LR_ = 7.5. Solid lines denote stable states, and dashed lines denote unstable states. (A) *M*_R_-*I*_R_ curves (top panel) and *M*_L_-*I*_R_ curves (bottom panel) are plotted for *g*_RR_ = 0 (black), 20 (red), 40 (green) and 60 (blue). Additional parameter is *τ_r_*_,RR_ = 463 ms. The values of *M*_R,c_ for *g*_RR_ = 40 and 60 are denoted by solid circles. (B) *M*_R_-*I*_R_ curves are plotted for *τ_r_*_.RR_ = 0 (black), 60 ms (red), 200 ms (green), 463 ms (blue) and 1000 ms (yellow). Additional parameter is *g*_RR_ = 40.
(EPS)

Figure S3 **Response of the RS-LTS network with RS-to-RS synaptic connections to step inputs** ***I*** **_R_Θ(** ***t*** **) to the RS neurons.** Time courses of *M*_R_ (top panel) and *M*_L_ (bottom panel) are shown. Additional parameters are *g*_RL_ = 35, g_LR_ = 7.5, g_RR_ = 40. Graphs on the right side present the same curves during the onset of activity with a shorter time scale.
(EPS)

Figure S4 **Fast cortical oscillations for large g_RR_.** Additional parameters are *g*_RL_ = 35, g_LR_ = 7.5. (A) Top: bifurcation diagram of the system in the *M*_R_-g_RR_ plane. Thin solid lines: stable fixed points; thin dotted line: unstable fixed points. Thick solid lines: minimum and maximum of *M*_R_ on stable limit cycles (periodic states). Open circles denote Hopf (HB) and saddle-node of periodics (SNP) bifurcation points. Bottom: the frequency *f* of the limit cycle plotted as a function of g_RR_. Additional parameter is *I*_R_ = 0.15. (B) Phase diagram of the system in the *I*_R_-g_RR_ plane. The fixed point is a stable state above the outer solid line, and the limit cycle is a stable state below the inner solid line. In the bistable grey area, both states are stable. (C) Traces of *M*_R_ (solid line) and *M*_L_ (dashed line) for g_RR_ = 60 and *I*_R_ = 0.15. LTS neurons fire almost exclusively during the periods when RS neuron fire.
(EPS)

Text S1 **Supplementary text.** RS-LTS networks: effects of firing-rate saturation and RS-to-RS recurrent connections.
(DOC)
