# Supplementary material for: LTS and FS Inhibitory Interneurons, Short-Term Synaptic Plasticity, and Cortical Circuit Dynamics
Source: PLoS Comput Biol. 2011 Oct 27;7(10):e1002248. doi: 10.1371/journal.pcbi.1002248 (PMC3203067; doi:10.1371/journal.pcbi.1002248)
Supplement: Text S1 — Supplementary text. RS-LTS networks: effects of firing-rate saturation and RS-to-RS recurrent connections. (DOC) [file pcbi.1002248.s005.doc]

**Supplementary Information Text S1**

Firing-Rate Saturation

Neurons exhibit refractoriness and their firing rates do not diverge in response to strong depolarizing input. In order to explore the effects of saturation on the circuit dynamics, we replace Equations 4-6 by

$M_{i}=M_{\max,i}\tanh_{+}\left\{\beta\left[I_{i}(t)+I_{\text{syn,}i}(t)-\theta_{i}\right]/M_{\max,i}\right\}$ (S1)

where tanh_+_(x) = tanh(x) for *x* ≥ 0 and 0 otherwise, and *I*_syn_,*_i_*(*t*) is the synaptic input current from all the neurons in the network. Since electrophysiological experiments usually do not drive neurons to their maximal rates, we use the following reasonable values: *M*_max,R_ = 80 Hz, *M*_max,L_ = 200 Hz, *M*_max,F_ = 300 Hz. For low firing rates, *M_i_* calculated from Equation S1 approaches the value calculated from Equations 4-6. For high firing rates, *M_i_* saturates at *M*_max_,*_i_*. As a result, the firing rates *M*_R_ and *M*_L_ are almost unchanged at low rate and are reduced at high rates as a result of saturation (Figure S1). The *M*_R_-*I*_R_ and *M*_L_-*I*_R_ curves are also shifted to the right as a result of LTS-to-RS inhibition when *M*_i_ is given by Equation S1. Therefore, whereas saturation affects the activity at high rates, the contribution of LTS neurons in preventing the circuit from reaching the over-activated regime is qualitatively similar without and with saturation.

RS-LTS Networks with RS-to-RS Recurrent Connections

Most RS-RS synapses, especially in sensory neocortex, exhibit short-term depression . We analyze the effects of RS-RS excitatory synaptic connections and their depression on the network dynamics by computing the *M*_R_-*I*_R_ and *M*_L_-*I*_R_ curves of the network for several values of *g*_RR_ (Figure S2A). If *g*_RR_ is large enough, the system exhibits a stable rest state only if the firing rate *M*_R_ is larger than a critical firing rate *M*_R,c_. Therefore, as happens without depression , RS neurons cannot fire at very low rates. The critical firing rate *M*_R,c_ increases with *g*_RR_. As in the case *g*_RR_ =0, LTS neurons strongly decrease the slope of the *M*_R_-*I*_R_ curve when they start to fire, and the slope increases with *I*_R_. At high rates, *M*_R_-*I*_R_ and *M*_L_-*I*_R_ are linear. To explain this linear behavior, we note that the steady-state solution for the RS-to-RS synapses is (Equations 1,2)

$s_{\text{RR}}=\frac{\tau_{s,\text{RR}} U_{\text{RR}} M_{\text{R}}}{1+\tau_{r,\text{RR}} U_{\text{RR}} M_{\text{R}}}$ (S2)

When *M*_R_ >> (*U*_RR_ *τ_r_*_,RR_)^-1^, $s_{\text{RR}}\approx\tau_{s,\text{RR}}/\tau_{r,\text{RR}}$ and Equation 4 becomes

$M_{\text{R}}\approx\beta_{\text{R}}(I_{\text{R},\text{ext}}+g_{\text{RR}}\tau_{s,\text{RR}}/\tau_{r,\text{RR}}-g_{\text{RL}}\tau_{s,\text{RL}}/\tau_{r,\text{RL}}-\theta_{\text{R}})$ (S3)

The steady-state RS-to-RS synaptic input is constant at high firing rates, *M*_R_, because of the depression. For a smaller recovery time from depression *τ_r_*_,RR_, the linear regime of the *M*_R_-*I*_R_ is obtained for higher *M*_R_ (Figure S2B). For low *τ_r_*_,RR_ , such as *τ_r_*_,RR_ = 60 ms, the *M*_R_-*I*_R_ curve exhibits two stable regimes, one just above *I*_R,th_ and a second one for high *M*_R_. Bistability is observed for a restricted *I*_R_ regime. For *τ_r_*_,RR_ = 0 and large *g*_RR_, stable resting activity is obtained in an *I*_R_ regime around *I*_R,th_. This resting state becomes unstable as *I*_R_ increases, and the firing rates of the neuronal population diverge to very large values. This divergence can be prevented in a more complicated model that describes saturation of the firing rates at large input values .

Strong-enough RS-to-RS synaptic connections may induce fast network oscillations in rate models and spiking-neuron models of excitatory and inhibitory neuronal populations. The oscillation time period is on the order of *τ_s_*, and is much smaller than the time scale of short-term synaptic plasticity. We find these oscillations when RS-LTS networks with strong-enough *g*_RR_, respond to step currents (Figure S3). During the oscillating episode, LTS and RS neurons fire almost in phase. Synaptic depression decreases the effective strength of the recurrent connections. Depression may stop these fast oscillations after an initial transient; the dynamics may then evolve on the longer time scale of synaptic plasticity, eventually settling into a resting steady state in a similar manner to the dynamics with *g*_RR_=0 (compare Figures S3 and 3A). In a restricted range of *g*_RR_, depression does not terminate the fast oscillations, which become an attractor (Figure S4A). The oscillatory state appears when *I*_R_ is just above threshold, and disappears above a certain level of *I*_R_. This state may be the only attractor or may, within a narrow range, coexist with a stable fixed point (Figure S4B). Within each cycle of the oscillations, RS neurons fire during a certain time interval and are quiescent afterwards (Figure S4C) until a new cycle of activity starts. As with the transient oscillatory episodes, LTS neurons are active only when RS neurons are active, i.e. the two populations fire nearly in phase. The peak of the LTS activity lags the peak of RS activity. The amplitudes of *M*_R_ (Figure S4A, top) and *M*_L_, as well as the duration of the active state of RS neurons (not shown), increase with *g*_RR_. As a result, the oscillation frequency decreases with *g*_RR_ (Figure S4A, bottom).

References

1. Tsodyks MV, Markram H (1997) The neural code between neocortical pyramidal neurons depends on neurotransmitter release probability. Proc Natl Acad Sci USA 94: 719-723.

2. Abbott LF, Varela JA, Sen K, Nelson SB (1997) Synaptic depression and cortical gain control. Science 275: 220-224.

3. Wang XJ (1999) Synaptic basis of cortical persistent activity: the importance of NMDA receptors to working memory. J Neurosci 19: 9587-9603.

4. Dayan P, Abbott LF (2005) Theoretical Neuroscience - Computational and Mathematical Modeling of Neural Systems. Cambridge, MA: The MIT Press.

5. Tsodyks MV, Skaggs WE, Sejnowski TJ, McNaughton BL (1997) Paradoxical effects of external modulation of inhibitory interneurons. J Neurosci 17: 4382-4388.

6. Wilson HR, Cowan JD (1972) Excitatory and inhibitory interactions in localized populations of model neurons. Biophys J 12: 1-24.

7. Hansel D, Mato G (2003) Asynchronous states and the emergence of synchrony in large networks of interacting excitatory and inhibitory neurons. Neural Comput 15: 1-56.
